# Parents’ or legal guardians’ beliefs and attitudes about childhood vaccination: a scoping review

**DOI:** 10.1590/0034-7167-2024-0126

**Published:** 2024-09-06

**Authors:** Mariana Mesquita de Oliveira Lima, Aline Oliveira Silveira, Ana Paula Sarmento Charão Aureliano, Hellen Cristina Costa Rocha, Luciana Melo de Moura, Sammya Rodrigues dos Santos

**Affiliations:** I Universidade de Brasília. Brasília, Distrito Federal, Brazil; II Escola Superior de Ciências da Saúde. Brasília, Distrito Federal, Brazil

**Keywords:** Vaccination, Immunization Programs, Legal Guardians, Parents, Child, Preschool, Vacunación, Programas de Inmunización, Tutores Legales, Padres, Preescolar

## Abstract

**Objective:**

to map scientific evidence about perceptions, beliefs, knowledge and attitudes of parents or legal guardians of children under 5 years of age regarding routine childhood vaccination.

**Methods:**

a scoping review, conducted in accordance with the JBI framework. The searches were carried out in the PubMed/MEDLINE, Web of Science, Scopus and LILACS databases. A total of 5,535 studies were returned and 77 were selected, which met the inclusion criteria.

**Results:**

perceptions related to interaction with healthcare professionals and services, with family organization and structure, with social interaction and public policies, cultural, religious and personal beliefs, knowledge about vaccination schedule, vaccination and immunization process and sources of information are the main factors mapped and which can positively or negatively influence parents’ or legal guardians’ attitudes towards vaccinating children.

**Conclusions:**

the findings allow us to identify factors related to parents’ perception and beliefs about childhood vaccination.

## RESUMEN

**Objetivo:**

mapear la evidencia científica sobre las percepciones, creencias, conocimientos y actitudes de padres o tutores legales de niños menores de 5 años respecto de la vacunación infantil rutinaria.

**Métodos:**

revisión del alcance, realizada de acuerdo con el marco del JBI. Las búsquedas se realizaron en las bases de datos PubMed/MEDLINE, Web of Science, Scopus y LILACS. Se devolvieron 5.535 estudios y se seleccionaron 77, que cumplieron con los criterios de inclusion.

**Resultados:**

percepciones relacionadas con la interacción con los profesionales y servicios de salud, con la organización y estructura familiar, con la interacción social y las políticas públicas, creencias culturales, religiosas y personales, el conocimiento sobre el calendario de vacunación, el proceso de vacunación e inmunización y las fuentes de información son los principales factores mapeados y que pueden influir positiva o negativamente en las actitudes de los padres o tutores legales hacia la vacunación de los niños.

**Conclusiones:**

los hallazgos permiten identificar factores relacionados con la percepción y creencias de los padres sobre la vacunación infantil.

## INTRODUCTION

Vaccination is recognized as one of the essential technologies that contributes to protecting health and increasing life expectancy at birth. It is recognized that, with the exception of drinking water, no other measure has had a similar impact on reducing morbidity and mortality in the population as compared to vaccines^(1)^. However, delay, refusal, or partial administration of vaccine doses by parents is a significant public health problem that threatens broad immunity and causes high morbidity and mortality^(2)^. Despite the recognized benefits, achieving high and homogeneous vaccination coverage across territories is a challenging aspect and involves tackling the determinants of vaccine hesitancy^(3)^.

In 2019, the World Health Organization (WHO) defined vaccine hesitancy as one of the main threat factors to global health^(4)^. This phenomenon can be described as reluctance or refusal to be vaccinated, despite the availability of vaccines^(3)^. Individuals who hesitate may present total rejection, late acceptance or accompanied by doubts about the process^(5)^. This problem can be influenced and increased by factors such as decreased confidence in the vaccination process, underestimation of the risks associated with vaccine-preventable diseases, in addition to limited accessibility and quality of services^(3)^.

Parents’ or legal guardians’ beliefs are an important intervening factor in the decision-making process related to childhood vaccination. They are individual and each person has their own internalized system, based on their genetic and social history, interactions with other people and the environment. In interactions, the emergence, refinement, solidification, confirmation and challenge of beliefs occur. They are shaped and substantially changed through interactions with others and with oneself, according to the context in which one lives^(6)^. In this regard, beliefs distinguish and unite people, as, through coexistence, they influence each other and, when shared, give identity to families and communities^(7)^.

Children are more vulnerable and dependent on their parents’ or legal guardians’ attitude and action to access vaccination. However, depending on their internalized beliefs, they may hesitate and put their lives, development and health in the short, medium and long term at risk, exposing them to different diseases and conditions for which vaccines are indicated^(8)^.

Data from the United Nations Children’s Fund (UNICEF) highlights that one in five children around the world has zero doses or is undervaccinated, which demonstrates that maintaining high rates of vaccination coverage among children is a major and complex global challenge^(9)^. The Immunization Agenda 2030 (IA, 2030), defined by WHO, sets out an ambitious and comprehensive global vision and strategy for vaccines and immunization for the decade 2021-2030. The AI 2030 goals are designed to inspire implementation action and support efforts to improve health security, universal health coverage, access and equity in immunization, and innovation^(10)^. This strategy plays a fundamental role in designing the Sustainable Development Goals (SDGs), especially SDG 3 – Ensure healthy lives and promote well-being for all at all ages. Due to the essentiality of vaccination for health and development, AI 2030 articulates and indirectly contributes to achieving the other 16 objectives^(11)^.

In the context of the Americas, the Pan American Health Organization (PAHO) shows great concern about vaccine adherence reduction, as data shows that the region is the second worst in the world in terms of vaccination coverage. Two countries stand out – Brazil and Mexico – as they account for more than 50% of children who have never received a dose of vaccine^(12)^.

The Center for Disease Control and Prevention (CDC) pointed out in the report, which demonstrates the Ten Great Public Health Achievements^(13)^, that vaccination programs contributed to the decline in mortality and morbidity from several infectious diseases; however, it must be stated that, in order to be successful in reducing vaccine-preventable disease prevalence and incidence, vaccination programs depend on a high level of absorption and adherence by the population.

Bearing in mind that the achievement of vaccination targets stipulated by each country’s immunization programs is threatened by various factors, whether social, political, economic, demographic, among others, mapping and understanding the determinants of parents’ perceptions of childhood vaccination and how they influence the decision-making process is extremely important to develop health strategies aimed at improving care with a view to eliminating and controlling vaccine-preventable diseases as well as protecting and promoting child and collective health.

## OBJECTIVE

To map scientific evidence about perceptions, beliefs, knowledge and attitudes of parents or legal guardians of children under 5 years of age regarding routine childhood vaccination.

## METHODS

### Ethical aspects

This work is a scoping review and, for this reason, does not require approval by the Research Ethics Committee (REC).

### Study design, period and place

This is a scoping review, developed in accordance with JBI methodology. It is a type of review that aims to systematically identify and map the breadth of available evidence on a given topic, field, concept or question, generally regardless of the source (i.e., primary research, reviews, non-empirical evidence). Scoping reviews, therefore, can clarify key concepts/definitions as well as identify key gaps and characteristics or factors related to a concept, including those related to methodological research^(14)^.

The Preferred Reporting Items for Scoping Reviews (PRISMA-ScR) ^(15)^ was followed to develop the research protocol. A preliminary search for previous reviews was carried out in the Open Science Framework and PROSPERO in March 2023, and no studies guided by the research question of this review were found. Registration was carried out in the Open Science Framework on April 19, 2023 (https://osf.io/by2mx).

To define the study question, the Population, Context and Concept (PCC) mnemonic structure was used, as proposed by JBI^(14)^. Thus, the following determinants of interest for the study were defined: Population (P): parents or legal guardians of children under 5 years of age; Concept (C): parents’ or legal guardians’ perceptions, beliefs, knowledge and vaccination attitudes; Context (C): routine childhood vaccination. Thus, the guiding question of this review was: what is the scientific evidence about perceptions, beliefs, knowledge and attitudes of parents or legal guardians of children under 5 years of age in relation to routine childhood vaccination?

### Inclusion and exclusion criteria

Studies that had as participants parents or legal guardians of children under 5 years of age, which addressed their beliefs, perceptions, attitudes and knowledge about routine childhood vaccination in this age group, within the scope of the public health system, in addition to original scientific articles, with both a qualitative and quantitative approach, and review articles were included.

Studies that included as participants pregnant mothers who did not have other children, formal caregivers (such as nannies), studies with children with comorbidities or hospitalized in health establishments, which exclusively involved populations living at a disadvantage, such as migrants and tribals, whose population was exclusively specific groups, such as adherents to religions or philosophies of life, in which the population was made up of individuals who have already suffered from vaccine-preventable diseases, in addition to studies with an exclusively rural population and exclusively with healthcare professionals, were excluded. As for concept, studies that only reported vaccination coverage without mentioning parents’ or legal guardians’ perceptions and beliefs and that addressed the impact of pro-vaccine interventions were excluded. Regarding the context, studies with a comparative focus between the period of the COVID-19 pandemic or outbreaks of vaccine-preventable diseases and other periods and studies focusing on pro- and anti-vaccine movements were not considered. Concerning study design, validation studies of research instruments and gray literature were excluded due to the characteristics of the concept and its wide exploration in scientific literature.

### Study selections earch

The search strategies were constructed, based on the review question, by the main reviewer with the assistance of a librarian who has experience in databases focused on the health area. The initial search strategy was composed of MeSH terms, namely: immunization programs; vaccination; parents; legal guardians; family; child, preschool; child; infant; newborn; perception; attitudes. Pilot testing was carried out in the PubMed/MEDLINE database. Based on the initial result, with the objective of refining and expanding the collection of relevant studies, a second search strategy was developed with uncontrolled expressions (childhood vaccination; health behaviors; vaccination decisions). The combination took place using Boolean descriptors such as “OR” and “AND”, The searches were carried out in March 2023 in the PubMed/MEDLINE, *Literatura Latino-Americana e do Caribe em Ciências da Saúde* (LILACS), Scopus and Web of Science databases, English or Portuguese languages were established as limits, due to reviewers’ limitations regarding other languages. No time limits were adopted and, as a search field, “title and abstract” was established. The final search strategy was presented in the search data.

**Figure 1:**
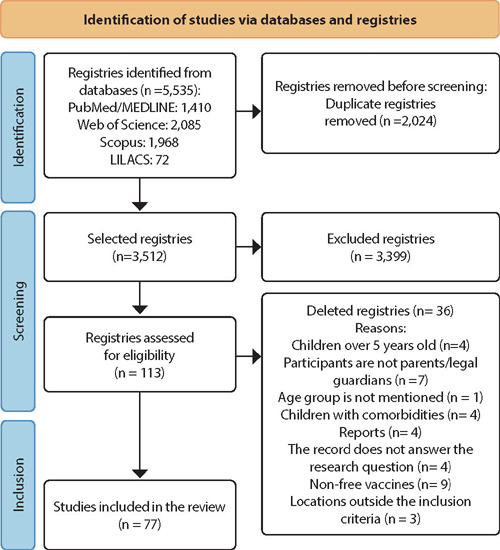
Flowchart of the selection and screening process of systematic review articles according to the PRISMA method^(16)^, 2024

The references found were exported to Rayyan” software (https://www.rayyan.ai/), available in a free online version, which helped in duplicate identification and exclusion and in article screening. Titles and abstracts were screened by two reviewers independently and blindly (double blind). References that were aligned with the research inclusion criteria were then classified as “included”, and those that were divergent, as “excluded” or “uncertain”, Disagreements among peers were resolved through discussion between reviewers or by decision of a third reviewer (expert) after assessing the highlighted article. Subsequently, the chosen studies were read in full by two independent reviewers, and disagreements were highlighted and discussed as a team until consensus was reached, proceeding to record the reasons for exclusion. This process was represented in the PRISMA flowchart^(16)^ (Figure 1).

Study methodological quality assessment was carried out using the instrument proposed by Hawker *et al.* ^(17)^, which consists of nine items: 1 = abstract and title; 2 = introduction and objectives; 3 = method and data; 4 = sampling; 5 = data analysis; 6 = ethics and prejudice; 7 = results; 8 = transferability or generalizability; and 9 = implications and usefulness. Each item is assessed on a four-grade scale (1 = very poor; 2 = poor; 3 = fair; 4 = good). Total scores ranged between 9 and 36, with the higher the score, the higher the quality. In this review, the methodological quality assessment aimed to demonstrate studies’ strengths and limitations, and the scores were not considered as an exclusion criterion. This process was carried out by two reviewers and checked by a third one.

### Data extraction, integration and synthesis of evidence

The JBI data extraction instrument^(14)^ was used at this time and, to organize the references in a systematic way, a file was created in Excel” software containing the items: study identification; article title; authors; complete reference; study location (institution, city, state and/or country); year of publication; research context; database; objective; study design and methodology; participant characteristics (sex, age, education, income, religion, occupation, sample size); main results; highlighted vaccines; study design; implications/recommendations; and methodological quality.

Data extraction was carried out by the main reviewer and later verified by another reviewer on the team. The data extraction form was identified with a sequential number of sources of evidence.

Study results were synthesized into two nuclei: descriptive information that favored childhood vaccination or limited it with the purpose of organizing it for the synthesis of evidence. The evidence was organized into thematic categories representing parents’ or legal guardians’ perceptions, beliefs, knowledge and attitudes in relation to childhood vaccination. These are determinants of the meanings attributed to childhood vaccination and the decision-making process regarding vaccination of children under 5 years of age.

## RESULTS

The search process resulted in 5,535 studies. After the first screening stage, 113 studies were considered eligible for full reading. Of these, 36 were excluded for not meeting the inclusion criteria, resulting in 77 articles that made up the final sample of this review, as illustrated in the PRISMA diagram^(16)^ (Figure 1).

The 77 studies were assessed for their methodological quality, reaching a maximum score of 36 points (n=4) and a minimum score of 24 points (n=2). Transferability, sampling and ethics were considered the greatest limitations of studies, according to Table 1, in research data.

### Characteristics of sources of evidence

Among the 77 studies included in this review (S1^18^ to S77^94^), siχ^(21, 36, 39, 40, 73, 83)^ are literature reviews, and the rest are primary studies, the majority of which are classified as quantitative studies (n=46), followed by qualitative studies (n=23) and two mixed-methods studies (Chart 1 – research data).

Concerning the temporal trend of publication on the topic, studies were published between 1987 and 2023, with a greater concentration in 2021 (n=10), 2019 (n= 9) and 2018 (n=7). In relation to their objectives, it is observed that the articles have in common the investigation of parents’ decision-making process in relation to childhood immunization, associating it with low local vaccination coverage and pointing out the determinants of vaccine hesitancy in this audience. Some studies focused on specific immunobiological investigations, such as the measles, mumps and rubella (MMR) vaccine (n=6), hepatitis B vaccine in newborns (n= 3) and rotavirus vaccine (n=2) due to controversies that arose at the time of publication involving these vaccines. From 2009 onwards, there was an intensification of interest in studying parents’ “knowledge, attitude and practice (KAP)” triad to understand possible barriers to vaccination acceptance in children under 5 years of age. When mapping the focus of study objectives, some main axes stand out, such as parents’ knowledge, attitudes, practices and beliefs (n=26), association between sociodemographic factors and vaccine hesitancy (n=7), guardians’ emotional factors (n=2) and immunobiological agent safety (n=2). The remaining articles aim to study children’s vaccination incompleteness comprehensively, seeking to determine and understand the various factors behind this phenomenon or identify characteristics that differentiate vaccinating parents from non-vaccinating parents (Chart 1 – Research Data).

**Figure 2:**
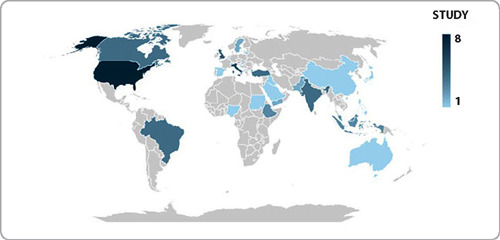
Distribution of article production worldwide, 2024

As for the country of publication, the review covers a global level, with studies produced in 31 different locations, with the highest production in the United States of America (n=8), Italy (n=6) and the United Kingdom (n=5), as illustrated in the world map below (Figure 2). To analyze the production of other countries, see the study characterization table (Chart 1 – Research Data).

In relation to family structure, all studies predominantly present married parents. As for guardians’ sex, 28 articles provided exclusively maternal data and 41 involved children’s father and mother, of which six addressed the participation of other guardians, such as grandparents. It was not possible to extract this data in eight studies, as they were literature reviews (n=6) or did not expose this information (n=2). It is noted that the participation of mothers is the majority, even in studies that reported the participation of both parents (Chart 1 – Research Data). Sample size varied between ten^(59)^ and 1,1206^(75)^ participants, with an age range of 15 to 49 years old.

From a socioeconomic point of view, studies included populations ranging from economically disadvantaged populations to participants with high financial income. This data varies depending on the economic reality, per capita income and financial stratification of each country. As for education, studies also addressed different levels, i.e., they ranged from illiterate (n=6) to parents/guardians with high school/higher education (n=37). Some studies cited the population’s occupation with the majority emplo yed^(25, 33, 44, 45, 52, 58, 65, 81)^, housewives^(19, 23, 26, 27, 35, 54)^, unemployed^(24, 31, 33)^, autonomous or informal^(22, 29)^ and specific profession^(28, 48)^ workers. The urban place of residence was predominant in relation to the rural one among the surveys, and in only four^(18, 26, 63, 66)^ the peasant population was higher than the city population. Some religions were mentioned by the articles, such as Hinduism^(18, 34)^, Muslims/Islam^(25, 33, 50, 63)^, Christians^(26, 28, 66)^ and Judaism^(38, 61, 63)^.

### Synthesis of evidence

Factors related to parents’ or legal guardians’ perceptions, knowledge and attitudes regarding vaccination of children under 5 years old were grouped into thematic categories (Chart 2), and beliefs (cultural, religious, personal) and individual experiences were subdivided into inhibitors and promoters of the childhood vaccination process (Chart 3).

**Chart 2:** Factors related to parents’ perceptions, knowledge and attitudes regarding vaccination, 2024

**Interaction with healthcare professionals**	Welcoming professionals^(26, 32, 61, 74, 82)^ and who promote relational security^(54, 82)^ are considered important by parents to feel confident in vaccinating their children^(53, 78)^. Professionals who talk openly and promote clear, complete and accessible information about the vaccine (immunological) and childhood vaccination and recommend it promote understanding and positively influence parents’ decisions, being considered sources of primary information by the majority^(18, 19, 20, 23, 26, 32, 33, 37, 38, 41, 43, 45, 49, 50, 51, 52, 57, 59, 60, 61, 62, 65, 66, 68, 69, 70, 72, 73, 76, 78, 85, 86, 88, 91)^, even before children are born^(81)^. Professionals who require childhood vaccination, but do not promote parents’ understanding of the benefits of immunization, achieve vaccination completeness, but do not promote healthy relationships with patients^(20, 41, 42)^. Professionals who do not dedicate enough time to talking to parents about childhood vaccination and the vaccination schedule clearly^(20, 21, 27, 39, 44, 47, 52, 53, 73, 77, 79, 83, 84, 91, 94)^, not very accessible and sensitive to parents’ demands^(21, 23, 32, 36, 39, 44, 45, 47, 49, 54, 56, 60, 71, 73, 74, 75, 82, 83, 85, 88, 90, 92)^, presenting hostile behavior^(47, 68, 73)^, language barrier^(21, 61)^, who pressure parents to vaccinate their children^(42, 56, 90)^ or do not recommend vaccination^(31, 44, 45, 52, 73, 91)^, which demonstrate limited and discordant knowledge on the subject^(32, 35, 44, 52, 54, 61, 85, 94)^, promote delays and rejection of children’s immunization.
**Knowledge about childhood vaccinations**	Parents who are aware of the childhood vaccination schedule^(26, 28, 32, 41, 43, 47, 48, 57, 70, 74, 78, 87, 91)^ and the vaccine-preventable disease^(45, 46, 65, 82)^, know where they can access vaccination^(43, 45)^ and consider that the benefits of vaccination outweigh the risks of adverse reactions^(19, 21, 22, 26, 28, 30, 42, 43, 44, 47, 48, 49, 50, 51, 56, 57, 63, 66, 69, 70, 76, 81, 87, 92, 93)^ are more likely to have a positive attitude^(22, 26, 27, 28, 32, 33, 43, 45, 46, 63, 65, 72, 73)^ towards vaccination. Parents who rely on the internet as reliable sources of information^(25, 31, 32, 33, 43, 50, 52, 56, 68, 72, 84, 85, 88)^, controversial medical literature^(20, 84, 88)^, media^(21, 31, 44, 46, 71, 75, 88, 90)^, pre-delivery groups^(88)^ and naturopaths/homeopaths^(43, 75, 84, 85)^ do not follow the recommendations proposed by the country’s immunization program. Parents who have limited knowledge about the childhood vaccination schedule^(21, 27, 35, 36, 45, 46, 48, 53, 57, 77, 83, 85)^, vaccine-preventable disease^(62, 65, 80, 87, 94)^, immunobiological^(22, 31, 36, 38, 45, 57, 62, 66)^/immunization process^(26, 27, 31, 70, 94)^, where and when to be vaccinated^(53)^ or the benefits of immunization^(28, 49)^ present limitations to fully vaccinating their children.
**Access to healthcare services universally and free of charge**	Free childhood vaccination^(42, 61, 71, 78, 80)^ and offered close to home^(26, 32)^, without the need to wait for long periods^(26)^, are considered factors that boost the immunization of minors. Inaccessible vaccination services^(24, 73)^, with little security^(49)^, of low quality^(21, 36)^, which are closed most of the time^(48, 49)^ where vaccine is unavailable^(21, 23, 27, 36, 39, 49, 66, 73, 77)^, far from parents’ homes^(18, 21, 26, 27, 36, 39, 48, 49, 73, 74, 77)^, with long waiting li nes^(21, 25, 27, 40, 49, 50, 73, 81, 91)^ and schedules that do not meet parents’ needs^(21, 39, 73, 91)^ are considered obstacles to childhood vaccination.
**Family organization dynamics and patterns**	Parents who plan to take their children to be vaccinated within their family routine have more complete vaccination records^(32)^. Parents who have several tasks and forget^(21, 27, 32, 39, 94)^ or do not have time to take their children to be vaccinated^(20, 23, 25, 32, 33, 40, 42, 50, 55, 56, 61, 73, 74, 77, 83, 94)^ and do not be involved^(21, 23, 27, 29, 42)^ to carry out the activity have difficulties in keeping their vaccination records up to date. Families who change states^(20, 53, 66, 94)^ or doctor’s offices^(20)^ face difficulties in vaccinating their children.
**Family structure**	Families with married parents, where there is extended family and community social support or a joint family structure^(18, 36, 50, 53, 53)^ and where the number of children is smaller^(30)^, favor vaccine hesitancy/vaccine refusal reduction. Mothers who have experienced postpartum depression^(20)^ and/or do not have family support^(53)^, within the patriarchal system^(21, 25, 31, 39)^ or are single mothers^(33, 39, 53)^ and have more children^(21, 24, 39, 52, 53, 91)^ face difficulties in vaccinating their children.
**Social interactions**	Family tradition^(20, 68)^, influence of friends and relatives^(22, 26, 42, 43, 45, 58, 62, 70, 94)^ and celebrities^(20)^ in favor of vaccination are considered motivating factors to vaccinate children under 5 years of age. The negative influence of friends, family^(20, 21, 25, 31, 37, 38, 42, 46, 49, 52, 56, 68, 71, 79, 83)^ and religious leaders^(25, 55)^ or politicians^(44, 53)^ in relation to childhood vaccination makes it difficult for parents to accept the practice.
**Public social and health policies – children’s social rights – right to life and health – and structure/operation of vaccination programs**	Parents who agree and trust the immunization policy of the country’s Ministry of Health are more willing to vaccinate their children with vaccines on the local schedule^(19, 23, 38, 42, 43, 44, 48, 49, 57, 63, 70)^. The mandatory vaccination of children in the country^(32, 41, 44, 46, 61)^ or for children’s entry into school life helps to increase vaccination rates^(19, 20, 37, 81)^. Parents who support governments^(49)^ and leaders who are in favor of childhood vaccination^(18)^ consider vaccination to be important for children’s health. Parents who consider the local national immunization program to be complete^(71)^ do not agree to vaccinate their children with new vaccines, whereas parents who perceive the vaccination schedule as strict^(47, 61, 67)^ are resistant to following it. Parents who are against the mandatory vaccination law in the country^(32, 44, 47, 68, 71)^ have disbelief in the government, regulatory agencies^(32, 50)^ and the local immunization program^(39, 47, 52, 60, 63, 67, 79, 82, 90)^, not being adherents to childhood vaccination.

**Chart 3:** Parental beliefs about childhood vaccination, 2024

**Beliefs that inhibit childhood vaccination**	Vaccination as an action that causes pain and suffering^(53, 61, 74, 80)^, trauma^(38, 88)^, immunological overload^(20, 26, 27, 32, 34, 40, 42, 44, 46, 49, 52, 67, 71, 75, 82, 84, 89, 90)^, risks to children’s health^(19, 20, 21, 22, 23, 24, 25, 26, 27, 29, 31, 32, 33, 34, 37, 39, 40, 41, 42, 43, 45, 46, 47, 48, 49, 50, 51, 52, 53, 54, 56, 57, 59, 61, 62, 65, 66, 67, 68, 70, 71, 72, 73, 74, 75, 79, 82, 83, 84, 85, 86, 87, 88, 89, 80, 91, 92, 93)^ Disbelief in vaccine effectiveness^(23, 31, 35, 38, 40, 44, 46, 51, 59, 62, 65, 71, 75, 85, 88, 90, 91, 92)^ and in western medicine^(23, 25, 38, 40, 51, 52, 60, 82, 85, 92)^. Insecurity, fear and uncertainty in relation to immunological products (pharmaceutical industry, tests)^(18, 20, 23, 24, 31, 32, 33, 35, 38, 40, 41, 42, 43, 44, 51, 52, 56, 59, 61, 62, 67, 71, 75, 79, 83, 84)^ and the childhood vaccination process^(37, 53, 79, 82, 85, 89)^. Beliefs in false contraindications to vaccination^(18, 20, 21, 29, 33, 48, 49, 50, 53, 57, 61, 71, 75, 82, 84, 94)^. Beliefs in naturally acquired immunity being better than vaccination^(24, 39, 40, 42, 46, 50, 59, 62, 67, 71, 79, 84, 90)^. Bad vaccination experiences with older children^(20, 21, 29, 35, 37, 44, 46, 54, 55, 56, 61, 66, 68, 71, 83)^. Beliefs that their children are too young to be vaccinated, based on cultural beliefs^(20, 32, 38, 39, 42, 47, 52, 59, 67, 71, 88)^ that the mother was immunized during pregnancy^(88)^ and do not need to vaccinate her child, that is not susceptible to contracting the vaccine-preventable disease^(20, 32, 35, 62, 67, 71, 79,82, 83, 85, 86, 92)^, that vaccine-preventable diseases are not common or serious^(19, 23, 24, 33, 34, 43, 59, 61, 62, 65, 67, 68, 71, 79, 82, 83, 84, 85, 88, 90, 92)^, that they can control their course by trusting in the quality of the local health system, and that unimmunized children do not pose a danger to the community^(31)^. Feeling of shame and criticism related to poverty by mothers^(39, 49)^. Beliefs in alternative methods to protect children against vaccine-preventable diseases^(79, 88)^, such as love^(67)^, breastfeeding^(20, 31, 38, 42, 47, 67, 71)^, food^(25, 35, 67, 71, 82)^, homeopathy^(67)^, herbal products^(31, 35, 38, 50, 52)^ and spiritual practices^(31)^. Religious beliefs that state that vaccination causes harm to children’s health, considering it a sin^(20, 21, 22, 25, 29, 35, 36, 39, 48, 50, 55, 61, 63, 64, 85)^.
**Beliefs that promote childhood vaccination**	Vaccinating children is a duty^(42, 43, 67, 69, 74, 81)^ and parental care to spare their children the pain, suffering and long-term impacts on health resulting from vaccine-preventable diseases^(20, 21, 26, 37, 42, 53, 56, 69, 89)^. Vaccination protects children from serious illness and mortality^(18, 19, 24, 26, 28, 32, 34, 37, 42, 43, 45, 46, 48, 51, 53, 57, 58, 59, 65, 69, 70, 80, 81, 85, 86, 88, 90)^ and communities^(19, 20, 46, 47, 52, 56, 79)^, and it is a decision made without stress, where children’s behaviors indicative of suffering are faced naturally^(51, 69, 74)^ and multiple injections as necessary and not harmful^(27, 48, 51, 57, 83, 91)^. Religious belief that vaccination is not prohibited by religion^(48, 57)^ and is supported by religious leaders^(18)^ motivates parents to vaccinate their children. Successful experiences in vaccinating older children^(36, 46, 55, 70, 81, 85, 88)^ and the belief in vaccination as a “cultural norm”^(42, 85)^ positively influences the vaccination of younger children. Personal belief in considering vaccination as necessary by parents who travel constantly^(42)^.

The evidence summarized in Chart 2 allows the identification of the factors most cited by the 77 eligible studies as motivators for childhood vaccination practice, namely adequate knowledge about childhood vaccination and positive interaction with healthcare professionals. These determinants are closely related to each other, since the information that parents have about the immunizing agent and the vaccination process, based on welcoming care, rich in scientific evidence and open to clarification of doubts with healthcare professionals, promote family confidence in childhood immunization which is translated into promoting beliefs that favor the activity, according to evidence in Chart 3.

Some studies did not identify a positive association between the level of knowledge and vaccination completion^(38, 47)^, i.e., parents claimed to have no knowledge on the subject despite having a favorable attitude towards childhood immunization. A case-control study^(64)^ found no significant differences between parents in the two groups (complete vaccination and incomplete vaccination) in terms of knowledge about vaccines, highlighting that the main mediating variables that increase the probability of parents completing their children’s vaccination are lack of academic training, low level of communicative health literacy (ability to understand the meaning of medical information, in order to interact with the medical environment), high positive attitudes towards vaccination, weak negative attitudes towards vaccination, weak negative attitudes towards mandatory vaccination, considering unofficial information from sources opposing vaccination as untrustworthy. Another article^(43)^ showed, as a result of multivariate analysis, that the determinants of parental intention were more closely related to how parents felt about their knowledge (i.e., feeling of being sufficiently informed about vaccination, knowledge of importance of vaccinating at 2 months) than about what they objectively knew (knowledge of vaccine-preventable diseases and vaccines administered at 2, 4 and 6 months of life).

Among the conditions that inhibit childhood vaccination, there is a prevalence of factors related to limited or inefficient interaction with healthcare professionals and beliefs, whether religious, cultural, personal or based on individual experiences that limit childhood immunization. To the extent that parents/legal guardians are not welcomed by healthcare professionals in their questions or do not receive the necessary informational support to make a decision in favor of vaccination, they tend to act in accordance with their beliefs, especially focused on the risk of immunobiological agents, and, as a result, may adopt behaviors that distance them from vaccination practice.

Studies show that, in these situations, parental protection behavior is present, but practiced in the wrong way: protecting *from* the vaccine and not *through* the vaccine. This phenomenon even permeates parents who have high levels of education and knowledge about vaccination, but with negative attitudes towards childhood immunization, demonstrating the power that belief exerts over the family’s decision-making process^(47, 67)^.

## DISCUSSION

According to the results present in this review, the quality of interaction between parents or legal guardians and healthcare professionals is the main mediating factor in the decision-making process in relation to childhood vaccination, according to participants’ perception.

This finding is in line with another study, in which it was found that many parents attribute the non-vaccination of their children (refusal or delay) to the way they are treated by professionals who work in clinics and vaccination services^(95)^. According to a literature review (overview), the interaction between patients and service providers is the cornerstone for maintaining confidence in vaccination. These professionals’ knowledge and attitudes about vaccines have already proven to be crucial in their own adherence to the vaccine, in their intention to recommend it and in their patients’ adherence to the vaccine^(96)^.

For this reason, finding a skeptical professional can strongly change people’s opinions or reinforce the idea that vaccination is unsafe, especially among those who already refuse it. The need to strengthen confidence in vaccines goes hand in hand with the need to improve communication skills with care seekers (children, parents, families and communities)^(95)^.

As healthcare professionals play a fundamental role in protecting and promoting children’s health and have a duty to inform people about vaccines and risks arising from vaccination coverage that does not meet the recommended goals, investment in ongoing education, communication and health literacy for these professionals^(95)^.

Strengthening Primary Care is, among other factors, the recognition that health production takes place between people and that it is necessary to improve the relationship established between Primary Healthcare (PHC) services, such as vaccination, with their users. It is essential that services are easily accessible to the population, that users are at the center of attention and that orientation to communities’ health needs is the basis for organizing services^(97)^. Furthermore, nurses, by identifying the various critical nodes that involve the process and by planning effective and targeted actions to overcome the problem of low vaccination coverage, highlight the importance of immunization as a basic measure for vaccine-preventable disease control, promoting the success of this strategy in community health as the main actor in the territory.

Confidence in vaccines and perception of vaccine risk are directly related to vaccine hesitancy. A study aiming to assess maternal vaccine hesitancy and its associated factors identified that variables such as high family income, good relationships with healthcare professionals, willingness to wait for vaccination and campaigns were associated with greater trust and lower perception of the risk of vaccines. On the other hand, deliberate delay or the decision not to vaccinate children and previous experience with adverse reactions to the vaccine were associated with lower confidence and greater perceived risk of vaccines. This study also highlights that healthcare providers, especially nurses, play an important role in addressing vaccine hesitancy by guiding vaccination through a trusting relationship^(98)^.

Despite the notable presence of nursing working in vaccination service coordination and implementation in PHC, in this review, there was limited recognition of this professional as a source of health information, with the focus being on the medical category (pediatricians) when mentioning the class of healthcare professionals.

The belief system (cultural, religious, personal) as well as parents’ or legal guardians’ individual experiences stand out in this review as a significant determinant in the attitude to vaccinate, postpone or refuse vaccination.

The Health Belief Model (HBM) can help understand the reasons that lead parents and/or guardians of children to accept or not accept immunobiological agents as health-related action depends on the simultaneous occurrence of three factors: sufficient motivation to make health issues salient or relevant; belief in susceptibility to a serious health problem or its sequelae, known as perceived threat; and the belief that following a specific health recommendation would be beneficial in reducing the perceived threat at a subjectively acceptable cost. Cost refers to perceived barriers that must be overcome to follow health recommendations^(7)^.

The popular interpretation of risk is not always based on a rational approach, but rather on an “uncertainty and ambiguity” approach, in which uncertainties remain even in the face of scientific evidence^(99)^.

Thus, adherence to vaccination is subject to social imagination, which greatly influences the propensity of a given group to be vaccinated or not. There are several factors that affect such a decision, such as confidence in the importance, safety and effectiveness of vaccines, as well as compatibility with individuals’ religious values. Such data were found in countries with higher percentages of agreement on the issues that vaccines are safe, important and effective, as they had a higher percentage of reports of having vaccinated their children^(100)^. Added to this is the population’s confidence in vaccines and their adherence, as a result of recent coexistence with vaccine-preventable diseases, as occurred in Africa, Latin America and India^(101)^.

Another aspect that is closely related to beliefs is vaccine hesitancy, defined as the delay in accepting or refusing vaccines, despite the availability of vaccination services^(5)^. A hesitant attitude towards childhood vaccinations means that some parents have doubts about the benefits of vaccines, worrying about their safety and questioning their need^(102)^. Therefore, high rates of vaccine hesitancy lead to low demand for vaccines and vaccination coverage^(5)^.

In line with the findings of this review, a study that analyzed, in scientific productions, the reasons that lead parents and families of children to vaccine hesitancy in the context of controlling vaccine-preventable diseases, found that these were related to lack of (knowledge) about vaccines (fake news, fear of adverse events, underestimation of vaccine-preventable disease lethality) and in(decision) and lifestyle (healthy habits, alternative medicine and religion)^(103)^.

This review mapped the inhibiting beliefs, among others, of immunological overload, risks to children’s health, disbelief in vaccine effectiveness, insecurity, fear and uncertainty in relation to immunological agents (pharmaceutical industry and tests). This evidence is corroborated by a vaccination survey carried out in 28 European countries in 2019, which revealed that approximately one tenth of the European population considered that vaccines are not rigorously tested before authorization. One-third believed that vaccines can overwhelm or weaken the immune system and that they can cause the disease they protect against, and almost half believed that vaccines can cause serious side effects. Furthermore, three configurations of beliefs were identified regarding the efficacy, safety and usefulness of the vaccine: the hesitant type (11%) is defined by the perception that vaccines are ineffective; the trusting type (59%) is defined by beliefs that vaccines are effective, safe, well-tested and useful; and the tradeoff type (29%) combines beliefs that vaccines are effective, well tested and useful, with perceptions of likely harm or risk. Vaccine-trusting and tradeoff types have similar vaccination histories, indicating the significant role of factors other than beliefs in inducing the behavior^(104)^.

A study that aimed to analyze the social representations of Brazilians hesitant about vaccination against COVID-19 concluded that the representations captured demonstrate their difficulty in discerning information reliability and a social imaginary of doubts and uncertainties. Thus, social representations about a given object or phenomenon are worldviews impregnated with sociocultural values and beliefs that are constructed throughout a life trajectory and are capable of impacting social health practices^(99)^.

In childhood vaccination, representations are also decisive, due to the diversity and multiplicity of doses of immunobiological agents recommended in the first year of life and the representation of this practice to parents as an overload and greater risk of adverse effects. In this scenario, parental omission bias, defined as being more averse to the risks associated with an action — taking an “unsafe” vaccine — than to the risks associated with inaction — running the risk of contracting a vaccine-preventable disease, plays a relevant role in individuals’ attitudes of rejecting or postponing vaccination^(96)^.

The review findings are related to the 3Cs model^(105)^ proposed by the WHO, especially with regard to the component of parental beliefs (Chart 3), as complacency (not perceiving diseases as high risk and vaccination as necessary) and trust (lack of confidence in vaccine safety and effectiveness) are the domains most influenced by them, resulting in parents’ hesitant behavior. In this context, therefore, health education can act effectively to demystify them, promoting clear knowledge based on scientific evidence^(95)^.

### Study limitations

This scoping review has as limitations non-inclusion of gray literature, restriction of languages (English and Portuguese) in the search for sources of evidence and non-inclusion of studies on campaign vaccination. It is important to conduct studies with rural populations and children with comorbidities, considering that these were not the populations considered as the focus of this review.

### Contributions to nursing, health or public policy

This review points out as practical recommendations training healthcare professionals regarding scientific knowledge about immunization and communication skills with the family in the context of childhood vaccination, with the aim of reducing the population’s fears and increasing confidence when carrying out health education. evidence-based. Timely parental counseling, emphasis on vaccination at the recommended age, and investigation of vaccination status can be performed by healthcare providers in any setting located in a healthcare facility, and parental awareness on the subject should ideally be done at the beginning of pregnancy. Regarding alternative vaccination schedules, healthcare providers need to be cautious and discuss the risks associated with delaying vaccinations when negotiating with parents about when and how childhood vaccinations can be completed.

The importance of including and deepening the topic of immunization in the educational system is highlighted, envisioning a potential impact on individual and community health, positioning individuals to be better prepared to manage information about their own health across the lifespan.

The findings suggest recommendations related to new research involving the role of nurses in childhood vaccination process, about personalized and well-designed media and educational campaigns as well as scientific work in remote and difficult-to-access areas. It is also recommended that studies be developed with a view to mapping parents’ perceptions and beliefs regarding childhood vaccination in the campaign strategy so that a comparison can be established between the two forms of collective health protection in terms of their determinants in guardians’ decision-making process and the meanings of such practice for this population.

Due to the wide range of individual and social determinants that drive or hinder vaccine acceptance in the pediatric population, it is recommended that local research be carried out to identify the main factors that affect the success of the immunization program in conjunction with regional characteristics and particularities, as the review aims to synthesize this evidence globally.

## CONCLUSIONS

This scoping review mapped a set of evidence on parents’ or legal guardians’ perceptions, beliefs, knowledge and attitudes about routine childhood vaccination as well as its related factors. According to parents’ perception, the main determinants for decision-making regarding childhood vaccination are: interaction with healthcare professionals; beliefs (cultural, religious, personal) and individual experiences; knowledge about childhood vaccinations; access to healthcare services universally and free of charge; dynamics and patterns of family organization; family structure; social interactions; and public social and health policies involving vaccination programs. These determinants influence parental meaning and behavior.

The representative evidence of parents’ perception allowed the identification of both inhibiting beliefs, generating suffering and fear, and promoting beliefs, based on parental duty and care, determining adherence to childhood vaccination. This knowledge supports nursing practice focused on the subjective and unique beliefs and needs of parents or legal guardians of children under 5 years of age in addressing vaccine hesitancy.

## Supplementary Material

0034-7167-reben-77-04-e20240126-suppl01

0034-7167-reben-77-04-e20240126-suppl02

## Data Availability

https://doi.org/10.48331/scielodata.VL93JN
