# ERRATA

**DOI:** 10.1590/0034-7167.20247706e06pt

**Published:** 2025-01-13

No artigo “Crenças e atitudes de pais ou responsáveis legais sobre a vacinação infantil: revisão de escopo”, com número DOI: https://doi.org/10.1590/0034-7167-2024-0126pt, publicado no periódico Revista Brasileira de Enfermagem, 2024;77(4):e20240126, página 5:

Onde se lia:

Alguns estudos não identificaram associação positiva entre o nível de conhecimento e a completude vacinal^(38^,^47)^, ou seja, os pais alegaram não ter conhecimento sobre a temática apesar de apresentarem atitude favorável em relação à imunização infantil. Estudo caso-controle^(64)^ não encontrou diferenças significativas entre os pais dos dois grupos (vacinação completa e vacinação incompleta) em termos de conhecimento sobre vacinas, destacando que as variáveis mediadoras principais que aumentam a probabilidade de os pais completarem a vacinação de seus filhos são ausência de formação acadêmica, baixo nível de literacia comunicativa em saúde (capacidade de compreender o significado da informação médica, de modo a interagir com o ambiente médico), atitudes positivas elevadas em relação à vacinação, atitudes negativas fracas em relação à vacinação, atitudes negativas fracas em relação à vacinação obrigatória, considerando informações não oficiais de fontes que se opõem à vacinação como confiáveis.

Lê-se:

Alguns estudos não identificaram associação positiva entre o nível de conhecimento e a completude vacinal^(38^,^47)^, ou seja, os pais alegaram não ter conhecimento sobre a temática apesar de apresentarem atitude favorável em relação à imunização infantil. Estudo caso-controle^(64)^ não encontrou diferenças significativas entre os pais dos dois grupos (vacinação completa e vacinação incompleta) em termos de conhecimento sobre vacinas, destacando que as variáveis mediadoras principais que aumentam a probabilidade de os pais completarem a vacinação de seus filhos são ausência de formação acadêmica, baixo nível de literacia comunicativa em saúde (capacidade de compreender o significado da informação médica, de modo a interagir com o ambiente médico), atitudes positivas elevadas em relação à vacinação, atitudes negativas fracas em relação à vacinação, atitudes negativas fracas em relação à vacinação obrigatória, considerando informações não oficiais de fontes que se opõem à vacinação como não confiáveis.

