# Supplementary material for: Parents’ or legal guardians’ beliefs and attitudes about childhood vaccination: a scoping review
Source: Rev Bras Enferm. 2024 Sep 6;77(4):e20240126. doi: 10.1590/0034-7167-2024-0126 (PMC11382679; doi:10.1590/0034-7167-2024-0126)
Supplement: 0034-7167-reben-77-04-e20240126-suppl01 [file 0034-7167-reben-77-04-e20240126-suppl01.pdf]

Estratégia de busca final

(("immunization programs"[Title/Abstract] OR "vaccination"[Title/Abstract] OR "childhood vaccination"[Title/Abstract] OR "child immunisation"[All Fields]) AND ("parent s"[All Fields] OR "parentally"[All Fields] OR "parentals"[All Fields] OR "parented"[All Fields] OR "parenting"[MeSH Terms] OR "parenting"[All Fields] OR "parents"[MeSH Terms] OR "parents"[All Fields] OR "parent"[All Fields] OR "parental"[All Fields] OR ("legal guardians"[MeSH Terms] OR ("legal"[All Fields] AND "guardians"[All Fields]) OR "legal guardians"[All Fields]) OR ("familialities"[All Fields] OR "familiality"[All Fields] OR "familially"[All Fields] OR "familials"[All Fields] OR "familie"[All Fields] OR "family"[MeSH Terms] OR "family"[All Fields] OR "familial"[All Fields] OR "families"[All Fields] OR "family s"[All Fields] OR "familys"[All Fields])) AND ("child, preschool"[MeSH Terms] OR ("child"[All Fields] AND "preschool"[All Fields]) OR "preschool child"[All Fields] OR ("child"[All Fields] AND "preschool"[All Fields]) OR "child preschool"[All Fields] OR ("child"[MeSH Terms] OR "child"[All Fields] OR "children"[All Fields] OR "child s"[All Fields] OR "children s"[All Fields] OR "childrens"[All Fields] OR "childs"[All Fields]) OR ("infant"[MeSH Terms] OR "infant"[All Fields] OR "infants"[All Fields] OR "infant s"[All Fields]) OR ("infant, newborn"[MeSH Terms] OR ("infant"[All Fields] AND "newborn"[All Fields]) OR "newborn infant"[All Fields] OR "baby"[All Fields] OR "infant"[MeSH Terms] OR "infant"[All Fields])) AND ("health belief"[Title/Abstract] OR "perception"[Title/Abstract] OR "health behaviours"[Title/Abstract] OR "vaccination decisions"[Title/Abstract] OR "attitudes"[Title/Abstract])) AND (fft[Filter]).

Tabela 1- Qualidade metodológica das evidências, 2024

Artigo	Resumo e título	Introdução e objetivos	Método e dados	Amostragem	Análise de dados	Ética e preconceito	Resultados	Transferibilidade ou generalização	Implicações e utilidade	Total
E1[18]	4	4	4	4	3	4	4	3	4	34
E2[19]	4	4	3	4	3	4	4	4	4	34
E3[20]	4	4	3	4	4	4	4	2	4	33
E4[21]	4	4	4	3	2	1	4	4	4	30
E5[22]	4	4	3	3	3	1	2	2	2	24
E6[23]	4	4	3	4	4	3	4	3	4	33
E7[24]	4	4	4	4	3	3	4	4	4	34
E8[25]	4	4	3	4	4	4	4	4	4	35
E9[26]	4	4	4	4	4	4	4	3	4	35
E10[27]	4	4	4	4	4	4	4	3	4	35
E11[28]	4	4	4	4	4	4	4	4	4	36
E12[29]	4	4	3	3	3	4	3	2	4	30
E13[30]	4	4	4	4	4	4	4	3	4	35
E14[31]	4	3	4	3	4	4	4	4	4	34
E15[32]	4	4	4	2	3	4	4	3	4	32
E16[33]	4	4	4	4	3	4	4	3	4	34
E17[34]	4	4	4	3	3	4	4	3	4	33
E18[35]	3	4	4	4	4	4	4	4	4	35
E19[36]	4	4	3	3	3	3	4	4	4	32
E20[37]	4	4	4	4	4	4	4	2	4	34
E21[38]	4	4	3	2	4	4	4	3	4	32
E22[39]	4	4	4	4	4	2	4	3	4	33
E23[40]	4	4	4	3	3	4	4	4	3	33
E24[41]	4	4	4	3	2	4	3	3	3	30
E25[42]	4	4	4	3	4	4	4	4	3	34

E26[43]	4	4	4	4	4	4	4	4	4	36
E27[44]	4	4	4	4	4	4	4	4	4	36
E28[45]	4	4	4	4	4	4	4	4	4	36
E29[46]	4	4	3	3	3	3	4	3	4	31
E30[47]	3	3	2	3	3	3	3	3	3	26
E31[48]	4	4	3	3	4	4	4	3	4	33
E32[49]	4	4	3	2	3	4	4	3	4	31
E33[50]	4	4	3	4	4	4	4	3	4	34
E34[51]	4	4	4	3	4	4	4	3	4	34
E35[52]	4	4	4	4	4	4	4	4	3	35
E36[53]	4	4	4	3	4	4	4	3	3	33
E37[54]	4	4	3	3	4	4	4	4	4	34
E38[55]	4	4	2	2	3	2	2	3	3	25
E39[56]	4	4	4	4	4	3	4	3	4	34
E40[57]	4	4	4	3	4	4	4	3	4	34
E41[58]	4	4	4	3	4	4	4	3	4	34
E42[59]	4	4	4	3	3	4	4	3	4	33
E43[60]	3	4	3	3	3	3	4	3	3	29
E44[61]	4	4	3	4	4	4	4	4	4	35
E45[62]	4	4	4	4	3	3	4	4	4	34
E46[63]	4	4	4	3	3	4	2	4	4	32
E47[64]	4	4	4	2	4	4	4	3	4	33
E48[65]	4	4	4	4	4	4	4	3	4	35
E49[66]	4	4	4	3	4	4	4	3	4	34
E50[67]	4	4	3	3	4	3	4	3	3	31
E51[68]	4	4	3	3	4	3	4	3	3	31
E52[69]	4	4	4	3	4	4	4	3	4	34
E53[70]	4	4	4	3	4	3	4	3	3	32
E54[71]	4	4	4	3	3	4	4	3	4	33
E55[72]	4	3	4	4	4	4	4	3	3	33
E56[73]	4	2	2	4	4	4	4	4	4	32
E57[74]	4	4	4	3	3	4	4	4	4	34
E58[75]	3	3	2	3	3	3	3	2	2	24
E59[76]	4	4	4	3	4	4	4	3	4	34
E60[77]	4	3	2	3	3	2	3	2	3	25
E61[78]	4	4	2	3	3	2	3	3	3	27
E62[79]	4	4	4	4	3	4	4	4	3	34
E63[80]	4	4	3	2	3	4	4	4	4	32
E64[81]	4	4	3	3	4	3	4	4	3	32
E65[82]	4	2	4	3	4	3	4	4	4	32
E66[83]	4	4	3	3	3	4	4	4	4	33
E67[84]	4	3	4	4	4	4	4	3	4	34
E68[85]	4	4	4	4	4	4	4	3	4	35
E69[86]	4	4	4	4	4	4	4	4	2	34
E70[87]	4	4	4	3	4	3	4	4	3	33
E71[88]	4	4	4	4	4	4	4	3	4	35
E72[89]	4	3	4	4	4	3	4	4	4	34
E73[90]	4	3	3	3	3	4	3	3	3	29
E74[91]	4	4	3	4	3	3	4	4	4	33
E75[92]	4	4	3	3	4	3	3	3	4	31
E76[93]	4	4	3	3	3	4	3	3	3	30
E77[94]	4	4	3	2	3	2	3	4	3	28

E – estudo

Quadro 1- Caracterização dos estudos, 2024

ID	Autores	País/ano	Objetivo	Metodologia/referencial	Participantes
**E1** [18]	Goruntla *et al.*	Índia/2023	Compreender adequadamente a prevalência da hesitação vacinal nas comunidades ou hospitais e os fatores responsáveis pela imunização incompleta de crianças menores de cinco anos na Índia.	Estudo quantitativo/ analítico transversal de base hospitalar.	574 mães.
**E2**[19]	Sahoo *et al.*	Índia/2023	Determinar a hesitação vacinal e a atitude em relação à vacinação entre cuidadores de crianças entre 6 meses a 5 anos que frequentaram a clínica de imunização.	Estudo quantitativo/transversal.	196 responsáveis/92,3 % dos participantes foram as mães e 7,7% foram pais ou avós.
**E3**[20]	Hsu *et al.*	EUA/2022	Explorar os fatores que levam às taxas de vacinação abaixo do ideal, entendendo por que os pais sem fortes crenças anti-vacinas não vacinam totalmente seus filhos.	Estudo qualitativo.	41 mães.
**E4**[21]	Balgovinda *et al.*	Mundial/2022	Explorar as percepções dos pais e profissionais de saúde em relação à imunização infantil, por meio de análise temática.	Revisão sistemática.	Não se aplica.
**E5**[22]	Sapriadi *et al.*	Indonésia/2022	Analisar os fatores associados com a decisão dos pais na vacinação contra sarampo e rubéola em crianças.	Estudo qualitativo descritivo com abordagem participativa.	32 pessoas entre mães e pais.
**E6**[23]	Sabahelzain *et al.*	Sudão/2022	Avaliar a hesitação vacinal contra o sarampo e caracterizar os seus determinantes entre os pais sudaneses.	Estudo quantitativo/ transversal.	500 pais/ maioria mães.
**E7**[24]	Akman *et al.*	Turquia/2022	Determinar as atitudes dos pais em relação à vacinação infantil e definir os fatores que afetam essas atitudes.	Estudo quantitativo/descritivo.	347 mães.
**E8**[25]	Krishnan *et al.*	Malásia/2022	Explorar os motivos da rejeição dos pais à vacinação infantil.	Estudo qualitativo.	27 pais/ maioria mães 66,7% eram mães).
**E9**[26]	Alebe *et al.*	Etiópia/2021	Avaliar o conhecimento, a atitude e as práticas dos pais em relação à imunização de crianças em Wadla Woreda, Nordeste da Etiópia, 2019.	Estudo quantitativo/ transversal de base comunitária.	418 responsáveis/ 388 mães (92,8%); 27 pais (6,5%); 3 outros (0,7%).
**E10**[27]	Saeed *et al.*	Paquistão/2021	Avaliar se as percepções dos pais prejudicaram o esforço do Paquistão para atingir as suas metas de imunização.	Estudo quantitativo/ transversal .	300 pais/ maioria mães (86,3%).
**E11**[28]	Oli *et al.*	Nigéria/2021	Determinar a opinião dos pais e cuidadores sobre a	Estudo quantitativo /transversal .	2400 entrevistados

			vacinação infantil de rotina em duas grandes cidades (Awka e Onitsha) do estado de Anambra, sudeste da Nigéria.		entre pais e mães/ 1209 (50,4%) eram pais e 1191(49,6%) eram mães.
**E12**[29]	Terzi *et al.*	Turquia/2021	Determinar as causas da recusa da vacina entre pais que recusam ou adiam a vacinação dos seus filhos em Giresun, Turquia.	Estudo quantitativo/ descritivo transversal.	54 pais/ maioria eram mães (n=43, 79,6%). Os pais (16,7%) ou ambos os pais (3,7%) foram entrevistados nos demais casos.
**E13**[30]	Voo *et al.*	Malásia/2021	Investigar como os fatores sociodemográficos estavam associados ao conhecimento, conscientização e hesitação sobre vacinas entre os pais no leste da Malásia.	Estudo quantitativo/ transversal.	405 pais/ maioria eram mães (95,8%).
**E14**[31]	Atasever *e t al.*	Turquia/2021	Investigar minuciosamente a noção de rejeição de vacinas abordada como um problema de saúde global.	Estudo qualitativo.	20 mães.
**E15**[32]	Musa *et al.*	Federação Bosnia e Herzegovina/ 2021	Identificar barreiras e motivadores dos pais para a vacinação infantil.	Estudo qualitativo/ transversal.	19 mães e 3 pais.
**E16**[33]	Mayerová *et al.*	Albânia/ 2021	Estimar a oportunidade da imunização infantil e a confiança na vacina associada à fonte sobre saúde e às características maternas, socioeconômicas e geográficas.	Estudo quantitativo/Pesquisa demográfica e de saúde.	2113 pesquisas aceitas com pares mães-filhos para a análise de oportunidade de vacinação; e 1795 pesquisas aceitas com pares mães-filhos para a análise de confiança na vacina.
**E17**[34]	Wagner *et al.*	Índia/2021	Explorar a relação entre fatores sociodemográficos e a hesitação vacinal, e como isso se manifesta no contexto do estado de vacinação completa.	Estudo quantitativo /transversal.	305 mães.
**E18**[35]	Çelik *et al.*	Turquia/2021	Compreensão das causas e origens que moldam as decisões das mães para alterar suas atitudes e comportamento em relação à vacinação.	Estudo qualitativo.	23 entrevistas/ 2 casais; 5 pais e 16 mães.
**E19**[36]	Galadima *et al.*	África/2020	Reunir evidências sobre os fatores que influenciam a adesão à vacinação infantil na África, bem como fornecer evidências para futuros investigadores no desenvolvimento, implementação e avaliação entre as populações	Revisão sistemática.	Não se aplica

			africanas que irão melhorar a adesão à vacinação infantil.		
**E20**(37)	Mossey *et al.*	Canadá, 2020	Compreender as experiências de pais e enfermeiros na tomada de decisões sobre imunização infantil, especificamente sarampo-caxumba-rubéola (MMR) e/ou difteria-tétano-coqueluche acelular (dTpa).	Estudo qualitativo/descrição interpretativa.	16 responsáveis, sendo um deles pai.
**E21**(38)	Fridman *et al.*	Israel/2020	Avaliar as informações dos pais, as atitudes em relação às diretrizes de saúde e as fontes de educação dos pais em relação à vacina contra o vírus da hepatite B (HBV), e para realizar uma pesquisa de acompanhamento para estimar a taxa de atualização da imunização e das práticas de saúde nesta população.	Estudo quantitativo/prospectivo controlado e pareado.	50 puérperas (grupo de estudo); 45 puérperas (grupo controle).
**E22**(39)	Bangura *et al.*	África Subsariana/ 2020	Identificar as barreiras individuais enfrentadas pelos pais/responsáveis, prestadores de cuidados e sistemas de sáude que dificultam a cobertura da vacinação infantil na África Subsariana.	Revisão sistemática.	Não se aplica.
**E23**(40)	Haroune *et al.*	Os estudos se concentraram entre Canadá, Austrália, EUA e Inglaterra. / 2020	Investigar os fatores que contribuem para a hesitação vacinal para a imunização infantil entre os pais.	Revisão sistemática.	Maioria mães.
**E24**(41)	Restivo *et al.*	Itália/2020	Investigar o conhecimento e a adesão à vacinação por parte dos pais de crianças que frequentam o Jardim de Infância em Palermo após a implementação da vacinação obrigatória pelo projeto 'Sportello Vaccinale'.	Estudo quantitativo /transversal.	95 responsáveis (89% dos questionários foram preenchidos pelas mães, 7% pelos pais e 3% por ambos).
**E25**(42)	McNeil *et al.*	Canadá/2019	Compreender a tomada de decisão materna sobre vacinação para bebês de até 24 meses.	Estudo qualitativo/ análise de conteúdo indutiva qualitativa.	1560 mães.
**E26**(43)	Dubé *et al.*	Canadá/2019	Avaliar o conhecimento, as atitudes e as crenças sobre a Hesitação Vacinal (HV) e a vacinação (conhecimento, atitude e crença) de uma grande e diversificada amostra de mães de recém-nascidos.	Estudo quantitativo/ transversal.	2.645 mães de recém-nascidos.

**E27**(44)	Bianco *et al.*	Itália/2019	Avaliar potenciais determinantes da hesitação vacinal entre pais de crianças do jardim de infância na Itália para sugerir estratégias que pudessem melhorar as taxas de vacinação infantil.	Estudo quantitativo/transversal.	575 pais/ maioria mães (80,3%).
**E28**(45)	Napolitano *et al.*	Itália/2019	Investigar o conhecimento, crenças e comportamentos sobre a infecção por rotavírus e a vacinação infantil relativa e para investigar seus determinantes em uma amostra de pais na Itália.	Estudo quantitativo/ transversal.	307 responsáveis/maioria (84,5%) mães.
**E29**(46)	Lúcia Ludvigh Cintulová	Eslováquia/2019	Analisar o impacto dos sentimentos emocionais nas decisões dos pais de rejeitar a vacinação.	Estudo quantitativo/transversal.	875 mães.
**E30**(47)	Romijnders *et al.*	Holanda/2019	Explorar semelhanças e diferenças em fatores que desempenham um papel na tomada de decisões e informações sobre a vacinação infantil que irão ajudar a apoiar a tomada de decisão informada dos pais sobre a vacinação infantil.	Estudo qualitativo.	12 entrevistas/ 75% eram mães; 25% eram pais.
**E31**(48)	Singh *et al.*	Malásia/2019	Avaliar e medir o conhecimento e a atitude das mães pós-natais em relação à vacinação infantil.	Estudo quantitativo/transversal.	200 mães pós-natais.
**E32**(49)	Geraldine Sabate Ridad	Filipinas/2019	Determinar as barreiras percebidas pelos entrevistados ao longo dos aspectos das barreiras pessoais, geográficas e sociais, crenças e mitos sobre vacinação, conhecimento e conscientização sobre o Programa Ampliado de Vacinação e sua relação com o nível de adesão dos entrevistados à imunização.	Estudo quantitativo descritivo.	352 responsáveis/ maioria eram mães (75,28%), sendo pais somente 24,72%.
**E33**(50)	Syiroj *et al.*	Indonésia/2019	Explorar os fatores subjacentes que explicam por que os pais indonésios escolhem não imunizar ou imunizar parcialmente seus filhos.	Estudo qualitativo.	16 responsáveis/ 11 mães, 2 pais, 2 avós e 1 entrevista com ambos os pais.
**E34**(51)	Šeškutė *et al.*	Lituânia/2018	Avaliar o conhecimento e as atitudes atuais das puérperas em relação à imunização infantil.	Estudo quantitativo/transversal.	300 puérperas.
**E35**(52)	Giambi *et al.*	Itália/2018	Estimar o grau de hesitação parental em relação à vacinação existente na	Estudo quantitativo/inquérito transversal.	3.130 responsáveis/maioria são mães.

			Itália e investigar os seus determinantes entre pais de crianças com idades compreendidas entre os 16 e os 36 meses.		
**E36**[53]	Dasgupta *et al.*	Índia/2018	Descobrir a proporção e os fatores que contribuem para a hesitação vacinal para vacinações infantis em favelas da cidade de Siliguri, Bengala Ocidental.	Estudo quantitativo/transversal comunitário.	194 mães.
**E37**[54]	Melissa L. Carrion	EUA/2018	Identificar as razões específicas e o(s) contexto(s) da decisão mais amplo(s) que destacaram a vacinação dos participantes.	Estudo qualitativo descritivo.	50 mães.
**E38**[55]	Yunitasari *et al.*	Indonésia/2018	Analisar fatores relacionados à vacinação em Madurês.	Estudo quantitativo transversal.	97 mães.
**E39**[56]	Hatoková *et al.*	Eslováquia/2018	Investigar as questões e as heurísticas que desempenham um papel na parentalidade da tomada de decisão sobre a vacinação das crianças.	Estudo qualitativo.	34 mães.
**E40**[57]	Fauzia Faraj Bamatraf; Mazin Ahmed Jawass	Iêmen/2018	Avaliar o conhecimento e as atitudes em relação imunização infantil entre pais iemenitas em Al-Cidade de Mukalla.	Estudo quantitativo/analítico transversal.	400 responsáveis/ 73 % eram mães e o restante (27%) eram pais.
**E41**[58]	Alshammari *et al.*	Arábia Saudita/2017	Avaliar as percepções e atitudes em relação à imunização infantil de rotina entre os pais sauditas.	Estudo quantitativo /transversal.	453 responsáveis /247 (54,5%,) eram mães e 206 (45,5%,) eram pais.
**E42**[59]	Sjögren *et al.*	Suécia/2017	Estudar as concepções dos pais sobre a infecção e vacinação por rotavírus usando o Quadro Consolidado para Pesquisa de Implementação, um modelo baseado em pesquisas anteriores sobre implementação em cuidados de saúde.	Estudo qualitativo/descritivo e uma abordagem fenomenográfica.	10 responsáveis/9 mães e 1 pai.
**E43**[60]	Attwell *et al.*	Austrália/2017	Entender o que há nos cuidados de saúde alopáticos – o sistema especializado por trás da vacinação – que leva alguns pais a desconfiar tanto de seus pontos de acessos (profissionais de saúde em particular) e o sistema como um todo.	Estudo qualitativo.	27 responsáveis/24 mães e 3 pais.

**E44**[61]	Kurup *et al.*	Singapura/2017	Explorar as percepções dos pais sobre a vacinação de seus filhos em Singapura para identificar lacunas na prática atual de saúde e, portanto, ajudar a desenvolver programas futuros para melhorar as experiências dos pais em relação a vacinação dos seus filhos.	Estudo qualitativo descritivo.	19 responsáveis/10 pais e 9 mães.
**E45**[62]	Ask *et al.*	Suécia/2017	Identificar por que os pais recusaram deixar seu filho ser vacinado ou não tinham certeza.	Estudo quantitativo/transversal.	1.063 responsáveis/83% eram mães, 14% pais e 1% foram os dois que responderam.
**E46**[63]	Ababu *et al.*	Etiópia/2017	Investigar os potenciais determinantes comportamentais e socioeconômicos da utilização dos serviços de imunização.	Estudo quantitativo/transversal de base comunitária.	2.328 responsáveis/2.174 (96,3%) eram mães.
**E47**[64]	Aharon *et al.*	Israel/2016	Avaliar a relação entre os níveis de Literância em Saúde e a adesão dos pais às vacinas recomendadas e determinar como o conhecimento, as crenças e as atitudes em relação às vacinas medeiam essa relação.	Estudo quantitativo/caso-controle estratificado com uma coorte retrospectiva.	731 responsáveis (entrevista aplicada a um dos pais; não menciona sexo).
**E48**[65]	Weiss *et al.*	Suíça/2016	Investigar como os padrões de atitudes dos pais estão ligados ao processo de tomada de decisão a favor ou contra a vacinação MMR.	Estudo quantitativo/transversal.	189 responsáveis/98% eram mães.
**E49**[66]	Negussie *et al.*	Etiópia/2016	Identificar fatores determinantes da imunização infantil incompleta no distrito de Arbegona, zona Sidama, sul da Etiópia.	Estudo misto.	548 responsáveis (183 casos e 365 controles) / 154 (84,6%) dos casos e 294 (81,2%) dos controles eram mães; 28 (15,3%) dos casos e 68 (18,7%) dos controles, o pai ou outro adulto no domicílio atuando como cuidador principal foi o respondente.
**E50**[67]	Márcia Thereza Couto; Carolina Luisa Alves Barbieri	Brasil/2015	Compreender as dimensões do cuidado parental acerca da (não) vacinação infantil em famílias de alta renda e escolaridade residentes no Município de São Paulo - SP.	Estudo qualitativo.	15 casais.

**E51**[68]	Carolina Luísa Alves Barbieri; Márcia Thereza Couto	Brasil/2015	O analisar aspectos socioculturais envolvidos no processo de tomada de decisão a favor ou contra a vacinação entre famílias de classe alta e com maior escolaridade.	Estudo qualitativo.	15 casais.
**E52**[69]	Harvey *et al.*	Reino Unido/2015	Compreender os fatores que influenciaram as decisões dos pais, os pontos de vista sobre o procedimento de imunização e as reações aos comportamentos de dor do bebê.	Estudo quantitativo.	45 responsáveis/41 mães e 4 pais.
**E53**[70]	Al-lela *et al.*	Iraque/2014	Avaliar a correlação entre conhecimentos-práticas dos pais e a completude da vacinação das crianças menores de 2 anos.	Estudo quantitativo/coorte retrospectivo e transversal prospectivo.	528 responsáveis.
**E54**[71]	Harmsen *et al.*	Holanda/2013	Obter mais informações sobre os fatores por detrás das decisões entre pais holandeses de vacinar parcialmente ou não vacinar seus filhos, a fim de conceber informações públicas e intervenções que ajudem os pais a tomar decisões que melhor sirvam os seus filhos e a comunidade em geral.	Estudo qualitativo/grupos focais on-line.	60 responsáveis (não mencionou sexo).
**E55**[72]	McCauley *et al.*	EUA/2012	Avaliar as escolhas autorelatadas pelos entrevistados para vacinar os seus filhos pequenos; conhecimentos, atitudes e crenças sobre vacinação; e comunicação com o fornecedor de vacinação do seu filho.	Estudo quantitativo.	1.500 mães/maioria eram mães.
**E56**[73]	Favina *et al.*	África, Ásia, América Latina, Médio Oriente e Europa/2012	Identificar as razões pelas quais as crianças pequenas elegíveis têm vacinações incompletas ou nenhuma vacinação em países pobres ou de rendimento médio.	Revisão de literatura cinzenta.	Não se aplica.
**E57**[74]	Figueiredo *et al.*	Brasil/2011	Descrever as experiências de famílias sobre imunização de crianças menores de dois anos, com base em relatos de famílias brasileiras.	Estudo qualitativo/ descritivo.	22 responsáveis/ 17 mães, 04 pais e 01 avó materna.
**E58**[75]	Smith *et al.*	EUA/2011	Avaliar a associação entre as crenças dos pais sobre as vacinas, a sua decisão de adiar ou recusar vacinas para os seus filhos e a cobertura vacinal das	Estudo quantitativo/inquérito de saúde.	11.206 responsáveis/99% eram pais (mulher/homem) ou avós.

			crianças de até 35 meses de idade.		
**E59**[76]	Coniglio *et al.*	Itália/2011	Explorar as questões que envolvem a aceitação ou não aceitação dos pais das vacinas recomendadas para crianças.	Estudo quantitativo/transversal.	1215 responsáveis/ 93% eram mães e 7% pais.
**E60**[77]	Abubakir A Ba'amer *et al.*	Iêmen/2010	Determinar a cobertura vacinal para crianças de 12 a 23 meses em Al Mukalla, distrito da cidade, e identificar as razões da não vacinação	Estudo misto.	210 responsáveis (não mencionou sexo).
**E61**[78]	Nisar *et al.*	Paquistão/2010	Avaliar o conhecimento, atitude e práticas das mães em relação imunização de crianças de um ano em Mawatch Goth, cidade de Kemari, Karachi, Paquistão	Estudo quantitativo/transversal.	209 mães.
**E62**[79]	Borràs *et al.*	Espanha/2009	Investigar o conhecimento dos pais sobre vacinas pediátricas na Catalunha.	Estudo quantitativo/retrospectivo e transversal.	630 responsáveis/ 87,62% eram mães; 10,80% eram pais; e 10 casos eram avós.
**E63**[80]	Logullo *et al.*	Brasil/2008	Avaliar o papel dos canais de comunicação e outros fatores de risco associados ao não recebimento da vacina contra o sarampo no momento certo na cidade de São Paulo, Brasil, até o ano 2000.	Estudo quantitativo/ caso-controle exploratório.	122 pais (não menciona sexo).
**E64**[81]	Marie Tarrant; Neil Thomson	China/ 2008	Explorar as imunizações infantis com uma amostra de pais de uma população onde as crianças têm elevada cobertura vacinal e identificar fatores que possam encorajar a adesão em outras populações.	Estudo qualitativo.	364 pais/maioria eram mães.
**E65**[82]	Rachel Elizabeth Casiday	Reino Unido/2007	Determinar a conceituação de risco, confiança e tomada de decisão na vacinação MMR.	Estudo qualitativo.	87 pais/ 77 mães e 10 pais.
**E66**[83]	Tickner *et al.*	Mundial/2006	Fornecer informações sobre a satisfação dos pais com o serviço de imunização, a compreensão pública da ciência da imunização e a confiança na medicina e nos profissionais de saúde, para descobrir áreas que requerem investigação mais aprofundada e para identificar estratégias para	Revisão da literatura.	Não se aplica.

			melhorar a adesão à vacinação infantil.		
**E67**[84]	Cassella *et al.*	Reino Unido/2006	Explorar as influências sociais e culturais e as crenças de saúde associadas à baixa adesão à MMR.	Estudo misto etnográfico.	452 mães.
**E68**[85]	Benin *et al.*	EUA/2006	Investigar a tomada de decisão sobre vacinações para bebês.	Estudo qualitativo.	33 mães.
**E69**[86]	Bigham *et al.*	Canadá/2006	Avaliar a cobertura de imunização contra hepatite B entre a primeira coorte de seis meses de crianças elegíveis na província fora da região de saúde de Vancouver-Richmond e avaliar os determinantes comportamentais e atitudinais dos pais/responsáveis da imunização contra hepatite B.	Estudo quantitativo, transversal.	487 pais (não menciona sexo).
**E70**[87]	Matsumura *et al.*	Japão/2005	Examinar a cobertura vacinal contra o sarampo entre crianças de 18 e 36 meses; o conhecimento e percepções dos pais sobre o sarampo e sua vacinação; e os fatores relacionados à vacinação incompleta contra o sarampo nas comunidades locais japonesas.	Estudo quantitativo, transversal.	2.707 mães no grupo de 18 meses e 2.340 mães no grupo de 36 meses.
**E71**[88]	Maayan-Metzgera *et al.*	Israel/2005	Identificar as características das mães que impedem que seus recém-nascidos recebam a vacina contra hepatite B.	Estudo quantitativo/caso-controle.	51 puérperas oponentes serviram como grupo de estudo; o grupo controle foi composto por 153 puérperas.
**E72**[89]	Bardenheier *et al.*	EUA/2004	Identificar as percepções dos pais em relação à segurança das vacinas e avaliar a sua relação com o estado de vacinação das crianças.	Estudo quantitativo/caso-controle.	2315 mães/ 1.016 eram casos e 1.299 eram controles.
**E73**[90]	Evans *et al.*	Grã-Betanha/2001	Investigar o que influencia as decisões dos pais sobre se aceitar ou recusar a imunização primária MMR e o impacto da recente controvérsia sobre sua segurança.	Estudo qualitativo.	48 pais (não menciona sexo).
**E74**[91]	Angelillo *et al.*	Itália/1999	Avaliar a conhecimento, atitudes e comportamento das mães italianas sobre a imunização dos bebês.	Estudo quantitativo/transversal.	841 mães.

**E75**[92]	Meszaros *et al.*	EUA/1996	Investigar os processos de decisão de alguns pais que optam por vacinar e de alguns pais que optam por não vacinar seus filhos.	Estudo quantitativo/transversal.	294 pais/ 94% eram mães.
**E76**[93]	Asch *et al.*	EUA/1994	Examinar o papel do viés de omissão nas decisões reais sobre vacinação.	Estudo quantitativo/transversal.	103 pais/ 99 mães e 4 pais.
**E77**[94]	Lakhani *et al.*	Reino Unido/ 1987	Entender os motivos pelos quais os pais, profissionais ou sistemas estão resultando em um baixo nível de adesão a vacina.	Estudo quantitativo/ caso-controle.	174 pais (não menciona sexo).

Id – identificação; E – estudo
