# Supplementary material for: Parents’ or legal guardians’ beliefs and attitudes about childhood vaccination: a scoping review
Source: Rev Bras Enferm. 2024 Sep 6;77(4):e20240126. doi: 10.1590/0034-7167-2024-0126 (PMC11382679; doi:10.1590/0034-7167-2024-0126)
Supplement: 0034-7167-reben-77-04-e20240126-suppl02 [file 0034-7167-reben-77-04-e20240126-suppl02.pdf]

Este arquivo README foi gerado em [2024-02-17] por [Mariana Lima]

INFORMAÇÕES GERAIS

Título do conjunto de dados: Percepções e crenças dos pais sobre a vacinação infantil e suas implicações: revisão do escopo

Informações do Autor/Investigador Principal

Nome: Mariana Lima

ORCID:0000-0002-0670-7782

Instituição: Universidade de Brasília, Brasil

Endereço: Brasília, Distrito Federal, Brasil

E-mail: mmos.df@gmail.com

Informações do autor/associado ou co-investigador

Nome: Aline Silveira

ORCID: 0000-0003-4470-7529

Instituição: Universidade de Brasília, Brasil

Endereço: Brasília, Distrito Federal, Brasil

E-mail: alinesilveira@unb.br

Autor/informações de contato alternativo

Nome: Ana Paula Aureliano

ORCID: 000-0001-9358-2174

Instituição: Universidade de Brasília, Brasil

Endereço: Brasília, Distrito Federal, Brasil

E-mail: charaoanapaula@gmail.com

Data de recolha de dados: 2023-03-22

Localização geográfica da coleta de dados: Brasília, Distrito Federal, Brasil

Informação sobre as fontes de financiamento que apoiaram a recolha dos dados: recursos próprios

VISÃO GERAL DE DADOS E ARQUIVOS

Lista de arquivos: estratégia de busca principal utilizada na base de dados PubMed/Medline, tabela de avaliação da qualidade metodológica das evidências selecionadas e tabela de caracterização dos estudos elegíveis da revisão de escopo.

Relacionamento entre arquivos: a estratégia de busca é um passo obrigatório para a seleção dos estudos que compõem a revisão e possibilitou o desenvolvimento do conteúdo contido nas tabelas.

NFORMAÇÕES METODOLÓGICAS

Descrição dos métodos utilizados para coleta/geração de dados: a revisão de escopo seguiu a metodologia descrita pelo manual do Joanna Briggs Institute (JBI) (Aromataris E, Munn Z. JBI Manual for Evidence Synthesis. Aromataris E, Munn Z, editors. JBI; 2020) e a avaliação da qualidade metodológica das evidências seguiu o instrumento proposto por Hawker et al (Hawker S, Payne S, Kerr C, Hardey M, Powell J. Appraising the evidence: Reviewing disparate data systematically. Qualitative Health Research. 2002 Nov;12(9):1284–99. DOI: 10.1177/1049732302238251).

Métodos de processamento dos dados: os dados foram extraídos das referências elegíveis de acordo com os critérios de inclusão e exclusão e tabelados no software Excel para o desenvolvimento das tabelas de acordo com os itens propostos pelo instrumento de avaliação de Hawker e da pergunta de pesquisa que direcionou a revisão. A estratégia de busca foi retirada da base de dados PubMed/Medline, após refinamento das estratégias testadas anteriormente.

Informações específicas do instrumento ou software necessárias para interpretar os dados: Microsoft® Excel® para Microsoft 365 MSO (Versão 2401 Build 16.0.17231.20194) 64 bits.

Pessoas envolvidas na coleta, processamento, análise e/ou envio de amostras: Mariana Lima, Aline Silveira, Ana Paula Aureliano, Hellen Rocha.
