# Supplementary material for: Parents’ or legal guardians’ beliefs and attitudes about childhood vaccination: a scoping review
Source: Rev Bras Enferm. 2024 Sep 6;77(4):e20240126. doi: 10.1590/0034-7167-2024-0126 (PMC11382679; doi:10.1590/0034-7167-2024-0126)
Supplement: Translation

# Translation: Crenças e atitudes de pais ou responsáveis legais sobre a vacinação infantil: revisão de escopo

**DOI:** 10.1590/0034-7167-2024-0126pt

**Authors:** Mariana Mesquita de Oliveira Lima, Aline Oliveira Silveira, Ana Paula Sarmento Charão Aureliano, Hellen Cristina Costa Rocha, Luciana Melo de Moura, Sammya Rodrigues dos Santos

**Affiliations:** I Universidade de Brasília. Brasília, Distrito Federal, Brasil; II Escola Superior de Ciências da Saúde. Brasília, Distrito Federal, Brasil

**Keywords:** Vacinação, Programas de Imunização, Tutores Legais, Pais, Pré-Escolar

## Abstract

**Objetivo:**

mapear as evidências científicas acerca das percepções, crenças, conhecimentos e atitudes de pais ou responsáveis legais de crianças menores de 5 anos quanto à vacinação infantil de rotina.

**Métodos:**

revisão de escopo, conduzida de acordo com o referencial do JBI. As buscas foram realizadas nas bases PubMed/MEDLINE, *Web of Science,* Scopus e LILACS. Retornaram 5.535 estudos e foram selecionados 77, que atenderam aos critérios de inclusão.

**Resultados:**

percepções relacionadas à interação com os profissionais e serviços de saúde, com a organização e estrutura familiar, com a interação social e políticas públicas, crenças culturais, religiosas e pessoais, conhecimentos sobre o calendário vacinal, o processo de vacinação e imunização e fontes de informação são os principais fatores mapeados e que podem influenciar positiva ou negativamente a atitudes dos pais ou responsáveis legais em vacinar crianças.

**Conclusões:**

os achados permitem identificar fatores relacionados à percepção e crenças dos pais sobre vacinação infantil.

## INTRODUÇÃO

A vacinação é consagrada como uma das tecnologias essenciais que contribui para proteger a saúde e aumentar a esperança de vida ao nascer. Reconhece-se que, com exceção da água potável, nenhuma outra medida teve um impacto semelhante na redução da morbimortalidade da população quanto às vacinas^(1)^. No entanto, o atraso, a recusa, ou a aplicação de doses parciais da vacina por parte dos pais são um problema significativo de saúde pública que ameaça a imunidade alargada e causa elevada morbidade e mortalidade^(2)^. Apesar dos reconhecidos benefícios, o alcance de altas e homogêneas coberturas vacinais nos territórios são aspectos desafiadores e envolvem o enfrentamento dos determinantes da hesitação vacina^(3)^.

A Organização Mundial da Saúde (OMS) definiu, em 2019, a hesitação vacinal como um dos principais fatores de ameaça à saúde global^(4)^. Esse fenômeno pode ser descrito como a relutância ou a recusa em vacinar-se, apesar da disponibilidade das vacinas^(3)^. O indivíduo que hesita pode apresentar rejeição total, aceitação tardia ou acompanhada de dúvidas sobre o processo^(5)^. Essa problemática pode ser influenciada e aumentada por fatores como diminuição da confiança no processo de vacinação, subestimação dos riscos associados a doenças evitáveis por vacinação, além da acessibilidade e qualidade limitadas dos serviços^(3)^.

As crenças dos pais ou responsáveis legais são um fator interveniente importante no processo de tomada de decisão relacionada à vacinação infantil. Elas são individuais e cada pessoa tem seu próprio sistema internalizado, baseado em sua história genética e social, nas interações com outras pessoas e com o meio ambiente. Nas interações, ocorrem o surgimento, o refinamento, a solidificação, a confirmação e o desafio das crenças. Elas são moldadas e substancialmente alteradas por meio das interações com os outros e consigo mesmo, de acordo com o contexto em que se vive^(6)^. Nesse sentido, as crenças distinguem e unem as pessoas, pois, mediante a convivência, influenciam uns aos outros e, quando partilhadas, conferem identidade às famílias e comunidades^(7)^.

As crianças são mais vulneráveis e dependentes da atitude e da ação dos pais ou responsáveis legais para terem acesso à vacinação. Contudo, esses, a depender das crenças internalizadas, podem hesitar e colocar em risco suas vidas, desenvolvimento e saúde a curto, médio e longo prazo, expondo-as a diferentes doenças e agravos para os quais as vacinas são indicadas^(8)^.

Os dados do Fundo das Nações Unidas para a Infância (UNICEF) destacam que uma em cada cinco crianças em todo o mundo está com dose zero ou subvacinada, o que demonstra que a manutenção de altas taxas de cobertura vacinal no público infantil é um grande e complexo desafio mundial^(9)^. A Agenda de Imunização 2030 (IA, 2030), definida pela OMS, estabelece uma visão e estratégia globais ambiciosas e abrangentes para vacinas e imunização para a década 2021-2030. Os objetivos da IA 2030 foram concebidos para inspirar ações de implementação e apoiar esforços para melhorar a segurança sanitária, a cobertura universal de saúde, o acesso e a equidade na imunização e na inovação^(10)^. Essa estratégia desempenha um papel fundamental na concepção dos Objetivos do Desenvolvimento Sustentável (ODS), especialmente o ODS 3 – Assegurar uma vida saudável e promover o bem-estar para todas e todos, em todas as idades. Devido à essencialidade da vacinação para a saúde e desenvolvimento, a IA 2030 articula-se e contribui indiretamente para o alcance dos outros 16 objetivos^(11)^.

No contexto das Américas, a Organização Pan-Americana da Saúde (OPAS) demonstra grande preocupação com a redução na adesão às vacinas, uma vez que os dados mostram que a região é a segunda pior no mundo em relação à cobertura vacinal. Dois países se destacam – Brasil e México -, pois respondem por mais de 50% das crianças que nunca receberam uma dose de vacina^(12)^.

O Centro de Controle e Prevenção de Doenças (CDC) apontou no relatório, que demonstra as Dez Grandes Conquistas da Saúde Pública^(13)^, que os programas de vacinação contribuíram para o declínio da mortalidade e morbidade de várias doenças infecciosas, porém não se pode deixar de afirmar que, para a obtenção do sucesso na redução da prevalência e incidência de doenças evitáveis por vacina, os programas de vacinação dependem de um alto nível de absorção e adesão por parte da população.

Tendo em vista que o alcance das metas vacinais estipuladas pelos programas de imunização de cada país sofre ameaças de diversos fatores, sejam de ordem social, política, econômica, demográfica, entre outros, mapear e compreender os determinantes nas percepções dos pais sobre a vacinação infantil e como eles influenciam o processo decisório é de extrema importância para o desenvolvimento de estratégias em saúde voltadas para a melhoria assistencial com vistas à eliminação e controle de doenças imunopreveníveis, além de proteção e promoção da saúde infantil e coletiva.

## OBJETIVO

Mapear as evidências científicas acerca das percepções, crenças, conhecimentos e atitudes de pais ou responsáveis legais de crianças menores de 5 anos quanto à vacinação infantil de rotina.

## MÉTODOS

### Aspectos éticos

O presente trabalho trata-se de revisão de escopo e, por esse motivo, não necessita de aprovação pelo Comitê de Ética em Pesquisa (CEP).

### Desenho, período e local do estudo

Revisão de escopo, desenvolvida de acordo com a metodologia do JBI. É um tipo de revisão que visa identificar e mapear sistematicamente a amplitude das evidências disponíveis sobre um determinado tópico, campo, conceito ou questão, geralmente independentemente da fonte (ou seja, pesquisa primária, revisões, evidências não empíricas). As revisões de escopo, portanto, podem esclarecer os principais conceitos/definições, assim como identificar as principais lacunas e características ou fatores relacionados a um conceito, incluindo aqueles relacionados à pesquisa metodológica^(14)^.

O *Preferred Reporting Items for Scoping Reviews* (PRISMA-ScR)^(15)^ foi seguido para o desenvolvimento do protocolo de pesquisa. Uma busca preliminar por revisões prévias foi realizada no *Open Science Framework* e no PROSPERO em março de 2023, e não foram encontrados estudos norteados pela pergunta de pesquisa desta revisão. O registro foi realizado no *Open Science Framework* em 19 de abril de 2023 (https://osf.io/by2mx).

Para definição da questão do estudo, foi utilizada a estrutura mnemônica População, Contexto e Conceito (PCC), conforme proposto pelo JBI^(14)^. Desse modo, foram definidos os seguintes determinantes de interesse do estudo: População (P): pais ou responsáveis legais de crianças menores de 5 anos de idade; Conceito (C): percepções, crenças, conhecimento e atitudes vacinais dos pais ou responsáveis legais; Contexto (C): vacinação infantil de rotina. Assim, a questão norteadora desta revisão foi: quais as evidências científicas acerca das percepções, crenças, conhecimentos e atitudes dos pais ou responsáveis legais de crianças menores de 5 anos em relação à vacinação infantil de rotina?

### Critérios de inclusão e exclusão

Foram incluídos estudos que tinham como participantes pais ou responsáveis legais de crianças menores de 5 anos, que abordassem suas crenças, percepções, atitudes e conhecimentos sobre a vacinação infantil de rotina nesta faixa etária, no âmbito do sistema público de saúde, além de artigos científicos originais, com abordagem tanto qualitativa quanto quantitativa, e artigos de revisão.

Foram excluídos estudos que incluíssem como participantes mães grávidas e que não tivessem outros filhos, cuidadores formais (como babás), estudos com crianças portadoras de comorbidades ou internadas em estabelecimentos de saúde, que envolvessem exclusivamente populações que vivessem em desvantagem, como migrantes e tribais, que tivessem como população, exclusivamente, grupos específicos, como adeptos a religiões ou filosofias de vida, em que a população fosse composta por indivíduos que já sofreram com doenças imunopreveníveis, além de estudos com população exclusivamente rural e exclusivamente com profissionais de saúde. Em relação ao conceito, foram excluídos estudos que só relatassem a cobertura vacinal sem mencionar as percepções e crenças dos pais ou responsáveis legais e que abordassem o impacto de intervenções pró-vacinais. Acerca do contexto, não foram considerados estudos com recorte comparativo entre o período da pandemia de COVID-19 ou surtos de doenças imunopreviníveis e outros períodos e estudos com foco em movimentos pró- e anti-vacina. Quanto ao tipo de estudo, excluíram-se estudos de validação de instrumentos de pesquisa e literatura cinzenta, devido às características do conceito e sua ampla exploração na literatura científica.

### Busca de seleção dos estudos

As estratégias de busca foram construídas, a partir da questão de revisão, pelo revisor principal com o auxílio de um bibliotecário que possui experiência em bases de dados voltadas para a área da saúde. A estratégia de busca inicial foi composta pelos MeSH *terms,* a saber: *immunization programs; vaccination; parents; legal guardians; family; child, preschool; child; infant; newborn; perception; attitudes.* O teste piloto foi realizado na base de dados PubMed/MEDLINE. A partir do resultado inicial, com o objetivo de refinamento e ampliação da captação de estudos relevantes, foi elaborada uma segunda estratégia de busca com expressões não controladas (*childhood vaccination;health behaviours; vaccination decisions*). A combinação se deu utilizando descritores booleanos como “*OR” e “AND”,* As buscas foram realizadas em março de 2023 nas bases PubMed/MEDLINE, Literatura Latino-Americana e do Caribe em Ciências da Saúde (LILACS), Scopus e *Web of Science,* estabelecendo-se como limites os idiomas inglês ou português, devido às limitações dos revisores quanto às outras línguas. Não foram adotados limites temporais e, como campo de busca, foi estabelecido “título e resumo”, A estratégia de busca final foi apresentada nos dados da pesquisa.

As referências encontradas foram exportadas para o *software* Rayyan® (https://www.rayyan.ai/), disponível em versão *online* gratuita, que auxiliou na identificação e exclusão das duplicatas e no processo de triagem dos artigos. Os títulos e resumos foram triados por dois revisores de forma independente e às cegas (duplo cego). As referências que estavam alinhadas aos critérios de inclusão da pesquisa foram então classificadas como “incluídas”, e as divergentes, como “excluídas” ou “incertas”, A resolução das divergências entre os pares foi feita por meio de discussão entre os revisores ou por decisão de um terceiro revisor (*expert*) após avaliação do artigo destacado. Posteriormente, os estudos escolhidos foram lidos integralmente por dois revisores independentes, e as discordâncias foram apontadas e discutidas em equipe até se alcançar o consenso, procedendo ao registro dos motivos de exclusão. Esse processo foi representado no fluxograma PRISMA^(16)^ (Figura 1).

**Figura 1:** Fluxograma do processo de seleção e triagem dos artigos de revisão sistemática segundo método PRISMA^(16)^, 2024

A avaliação de qualidade metodológica dos estudos foi realizada utilizando o instrumento proposto por Hawker *et* al.^(17)^, que consiste em nove itens: 1 = resumo e título; 2 = introdução e objetivos; 3 = método e dados; 4 = amostragem; 5 = análise de dados; 6 = ética e preconceito; 7 = resultados; 8 = transferibilidade ou generalização; e 9 = implicações e utilidade. Cada item é avaliado com escala de quatro graus (1 = muito ruim; 2 = ruim; 3 = regular; 4 = bom). Os escores totais variaram entre 9 e 36, sendo que, quanto maior a pontuação, maior a qualidade. Nesta revisão, a avaliação de qualidade metodológica teve como objetivo demonstrar forças e limitações dos estudos, e os escores não foram considerados como critério de exclusão. Esse processo foi realizado por dois revisores e conferido por um terceiro.

### Extração dos dados, integração e síntese das evidências

O instrumento de extração de dados do JBI^(14)^ foi utilizado neste momento e, para a organização das referências de forma sistemática, elaborou-se um fichamento no *software Excel*® contendo os itens: identificação do estudo; título do artigo; autores; referência completa; local de realização do estudo (instituição, cidade, estado e/ou país); ano de publicação; contexto da pesquisa; base de dados; objetivo; tipo de estudo e metodologia; características dos participantes (sexo, idade, escolaridade, renda, religião, ocupação, tamanho da amostra); principais resultados; vacinas destacadas; delineamento do estudo; implicações/recomendações; e qualidade metodológica.

A extração dos dados foi realizada pelo revisor principal e verificada, posteriormente, por outro revisor da equipe. A ficha de extração de dados foi identificada com um número sequencial das fontes de evidências.

Os resultados dos estudos foram sintetizados em dois núcleos: informações descritivas que favoreciam a vacinação infantil ou a limitavam com a finalidade de organizá-las para a síntese das evidências. As evidências foram organizadas em categorias temáticas representativas das percepções, crenças, conhecimentos e atitudes dos pais ou responsáveis legais em relação à vacinação infantil. Esses são determinantes dos significados atribuídos à vacinação infantil e do processo de tomada de decisão quanto à vacinação da criança menor de 5 anos.

## RESULTADOS

O processo de busca resultou em 5.535 estudos. Após a primeira etapa de triagem, foram considerados elegíveis para leitura na íntegra 113 estudos. Desses, 36 foram excluídos por não atenderem aos critérios de inclusão, resultando em 77 artigos que compuseram a amostra final desta revisão, conforme ilustrado no diagrama PRISMA^(16)^ (Figura 1).

Os 77 estudos foram avaliados quanto à sua qualidade metodológica, alcançando pontuação máxima de 36 pontos (n=4) e mínima de 24 pontos (n=2). A transferibilidade, a amostragem e a ética foram consideradas as maiores limitações dos estudos, de acordo com a Tabela 1, em Dados da Pesquisa.

### Características das fontes de evidências

Entre os 77 estudos incluídos nesta revisão (E1^18^ a E77^94^), seis ^(21, 36, 39, 40, 73, 83)^ são revisões de literatura, e o restante, estudos primários, sendo a maioria classificada como estudos quantitativos (n=46), seguida por estudos qualitativos (n=23) e dois mistos (Quadro 1 – Dados da Pesquisa).

No que se refere à tendência temporal de publicação sobre a temática, os estudos foram publicados entre os anos 1987 e 2023, apresentando maior concentração em 2021 (n=10), 2019 (n= 9) e 2018 (n=7). Em relação aos seus objetivos, observa-se que os artigos possuem em comum a investigação do processo decisório dos pais em relação à imunização infantil, associando-o à baixa cobertura vacinal local e apontando os determinantes da hesitação vacinal nesse público. Alguns estudos tiveram como foco de investigação imunobiológicos específicos, como a vacina contra sarampo, caxumba e rubéola (MMR) (n= 6), contra hepatite tipo B em recém-nascidos (n= 3) e contra o rotavírus (n=2), em virtude das controvérsias que surgiram à época da publicação envolvendo essas vacinas. A partir de 2009, verificou-se a intensificação do interesse em estudar a tríade “conhecimento, atitude e prática (CAP)” dos pais para a compreensão de possíveis barreiras à aceitação da vacinação em crianças menores de 5 anos. Ao mapear o foco dos objetivos dos estudos, destacam-se alguns eixos principais, como o conhecimento, as atitudes, as práticas e as crenças dos pais (n=26), a associação entre fatores sociode-mográficos com a hesitação vacinal (n=7), fatores emocionais dos responsáveis (n=2) e a segurança do imunobiológico (n=2). Os demais artigos têm o propósito de estudar a incompletude vacinal das crianças de forma mais ampla, buscando determinar e compreender os diversos fatores por trás desse fenômeno ou identificar características que diferenciam os pais vacinadores dos não vacinadores (Quadro 1 – Dados da Pesquisa).

Quanto ao país de publicação, a revisão abrange em nível mundial, com estudos produzidos em 31 localidades diferentes, com maior produção nos Estados Unidos da América (n=8), Itália (n=6) e Reino Unido (n=5), conforme ilustrado no mapa mundo abaixo (Figura 2). Para analisar a produção dos demais países, ver tabela de caracterização dos estudos (Quadro 1 – Dados da Pesquisa).

**Figura 2:** Distribuição da produção de artigos mundialmente, 2024

Em relação à estrutura familiar, todos os estudos apresentam predominantemente pais casados. Quanto ao sexo dos responsáveis, 28 artigos trouxeram os dados exclusivamente maternos e 41 envolviam o pai e a mãe da criança, sendo que, desses, seis abordaram a participação de outros responsáveis, como avós. Não foi possível a extração desse dado em oito publicações, pois tratavam-se de revisões de literatura (n=6) ou não expuseram essa informação (n=2). Nota-se que a participação das mães é majoritária, mesmo nos estudos que relataram a participação de ambos os pais (Quadro 1 – dados da pesquisa). O tamanho da amostra variou entre dez^(59)^ e 1.1206^(75)^ participantes, com faixa etária de 15 a 49 anos de idade.

Do ponto de vista socioeconómico, os estudos incluíram desde populações economicamente desfavorecidas até participantes que apresentavam alta renda financeira. Esse dado possui variações em decorrência da realidade económica, renda *per capita* e estratificação financeira de cada país. Quanto à escolaridade, as publicações também abordaram diferentes níveis, ou seja, incluíram desde analfabetos (n=6) a pais/responsáveis com ensino médio/superior (n= 37). Alguns estudos citaram a ocupação da população com maioria empregada^(25, 33, 44, 45, 52, 58, 65, 81)^, donas de casa^(19, 23, 26, 27, 35, 54)^, desempregada^(24, 31, 33)^, autónoma ou informal^(22, 29)^ e profissão específica^(28, 48)^. O local de moradia urbano foi predominante em relação ao rural entre as pesquisas, sendo que em apenas quatro^(18, 26, 63, 66)^ a população camponesa foi superior à população das cidades. Algumas religiões foram mencionadas pelos artigos, como hinduísmo^(18, 34)^, mulçumanos/islamismo^(25, 33, 50, 63)^, cristãos^(26, 28, 66)^ e judaísmo^(38, 61, 63)^.

### Síntese das evidências

Os fatores relacionados às percepções, crenças, conhecimentos e atitudes dos pais ou responsáveis legais em relação à vacinação de crianças menores de 5 anos foram agrupados em categorias temáticas (Quadro 2), e as crenças (culturais, religiosas, pessoais) e experiências individuais foram subdivididas em inibidoras e promotoras do processo de vacinação infantil (Quadro 3).

**Quadro 2:** Fatores relacionados às percepções, conhecimentos e atitudes dos pais em relação à vacinação, 2024

**Interação com os profissionais de saúde**	Profissionais acolhedores^(26, 32, 61, 74, 82)^ e que promovem segurança relacional^(54, 82)^ são considerados importantes pelos pais para sentirem-se confiantes na vacinação da criança^(53, 78)^. Profissionais que conversam abertamente e promovem informações claras, completas e acessíveis sobre a vacina (imunológico) e a vacinação infantil e a recomenda promovem a compreensão e influenciam positivamente a decisão dos pais, sendo considerados fontes de informação primária pela maioria^(18, 19, 20, 23, 26, 32, 33, 37, 38, 41, 43, 45, 49, 50, 51, 52, 57, 59, 60, 61, 62, 65, 66, 68, 69, 70, 72, 73, 76, 78, 85, 86, 88, 91)^, mesmo antes da criança nascer^(81)^. Profissionais que exigem a vacinação infantil, porém não promovem a compreensão dos pais sobre os benefícios da imunização, alcançam a completude vacinal, mas não favorecem relações saudáveis com os pacientes^(20, 41, 42)^. Profissionais que não dedicam tempo o bastante para conversar com os pais sobre a vacinação infantil e o calendário vacinal de forma clara^(20, 21, 27, 39, 44, 47, 52, 53, 73, 77, 79, 83, 84, 91, 94)^, pouco acessíveis e sensíveis às demandas dos pais^(21, 23, 32, 36, 39, 44, 45, 47, 49, 54, 56, 60, 71, 73, 74, 75, 82, 83, 85, 88, 90, 92)^, que apresentam comportamento hostil^(47, 68, 73)^, que possuem barreira linguística^(21, 61)^, que pressionam os pais para vacinarem seus filhos^(42, 56, 90)^ ou não indicam a vacinação^(31, 44, 45, 52, 73, 91)^, que demonstram conhecimento limitado e discordante sobre o assunto^(32, 35, 44, 52, 54, 61, 85, 94)^, promovem atrasos e rejeição à imunização das crianças.
**Conhecimentos sobre vacinação infantil**	Pais que têm conhecimento sobre o calendário de vacinação infantil^(26, 28, 32, 41, 43, 47, 48, 57, 70, 74, 78, 87, 91)^ e a doença imunoprevenível^(45, 46, 65, 82)^, sabem onde podem ter acesso à vacinação^(43, 45)^ e consideram que os benefícios da vacinação superam os riscos das reações adversas^(19, 21, 22, 26, 28, 30, 42, 43, 44, 47, 48, 49, 50, 51, 56, 57, 63, 66, 69, 70, 76, 81, 87, 92, 93)^ são mais favoráveis a terem uma atitude positiva^(22, 26, 27, 28, 32, 33, 43, 45, 46, 63, 65, 72, 73)^ em relação à vacinação. Pais que têm como fontes confiáveis de informação a internet^(25, 31, 32, 33, 43, 50, 52, 56, 68, 72, 84, 85, 88)^, literatura médica controversa^(20, 84, 88)^, mídia^(21, 31, 44, 46, 71, 75, 88, 90)^, grupos pré-parto^(88)^ e naturopatas/homeopatas^(43, 75, 84, 85)^ não seguem as recomendações propostas pelo programa de imunização do país. Pais que possuem conhecimento limitado sobre o calendário de vacinação infantil^(21, 27, 35, 36, 45, 46, 48, 53, 57, 77, 83, 85)^, sobre a doença imunoprevenível^(62, 65, 80, 87, 94)^, sobre o imunobiológico^(22, 31, 36, 38, 45, 57, 62, 66)^/processo de imunização^(26, 27, 31, 70, 94)^, sobre onde e quando se vacinar^(53)^ ou sobre os benefícios da imunização^(28, 49)^ apresentam limitações para vacinar completamente seus filhos.
**Acesso aos serviços de saúde de forma universal e gratuita**	Vacinação infantil gratuita^(42, 61, 71, 78, 80)^ e ofertada próxima ao domicílio^(26, 32)^, sem necessidade de esperar por longos períodos^(26)^, são considerados fatores que impulsionam a imunização dos menores. Serviços de vacinação inacessíveis^(24, 73)^, com pouca segurança^(49)^, de baixa qualidade^(21, 36)^, que encontram-se fechados na maioria do tempo^(48, 49)^, onde a vacina está indisponível^(21, 23, 27, 36, 39, 49, 66, 73, 77)^, localizados longe das residências dos pais^(18, 21, 26, 27, 36, 39, 48, 49, 73, 74, 77)^, com longas filas de espera^(21, 25, 27, 40, 49, 50, 73, 81, 91)^ e com horários que não atendem às necessidades dos pais^(21, 39, 73, 91)^, são considerados empecilhos para a vacinação infantil.
**Dinâmica e padrões de organização familiar**	Pais que se programam dentro da rotina familiar para levarem seus filhos para se vacinarem apresentam cadernetas de vacinação mais completas^(32)^. Pais que possuem vários afazeres e esquecem^(21, 27, 32, 39, 94)^ ou não têm tempo para levar os filhos para se vacinarem^(20, 23, 25, 32, 33, 40, 42, 50, 55, 56, 61, 73, 74, 77, 83, 94)^ e não se envolvem^(21, 23, 27, 29, 42)^ para desempenhar a atividade apresentam dificuldades em manter a caderneta de vacinação em dia. Famílias que mudam de estado^(20, 53, 66, 94)^ ou de consultório médico^(20)^ encaram dificuldades em vacinar seus filhos.
**Estrutura familiar**	Famílias com pais casados, onde existe apoio social familiar ampliado e comunitário ou estrutura familiar conjunta^(18, 36, 50, 53, 55)^ e que o número de filhos é menor^(30)^, favorecem a diminuição da hesitação vacinal/recusa vacinal. Mães que passaram por depressão pós-parto^(20)^ e/ou não contam com o apoio familiar^(53)^, dentro do sistema patriarcal^(21, 25, 31, 39)^ ou são mães solteiras^(33, 39, 53)^ e possuem mais filhos^(21, 24, 39, 52, 53, 91)^ enfrentam dificuldades em vacinar seus filhos.
**Interações sociais**	Tradição familiar^(20, 68)^, influência de amigos e parentes^(22, 26, 42, 43, 45, 58, 62, 70, 94)^ e celebridades^(20)^ a favor da vacinação são considerados fatores motivadores para vacinar crianças menores de 5 anos. A influência negativa de amigos, familiares^(20, 21, 25, 31, 37, 38, 42, 46, 49, 52, 56, 68, 71, 79, 83)^ e líderes religiosos^(25, 55)^ ou políticos^(^44,55^)^ em relação à vacinação infantil dificulta a aceitação da prática pelos pais.
**Políticas públicas sociais e de saúde – direitos sociais da criança – direito à vida e a saúde – e estrutura/funcionamento dos programas de vacinação**	Pais que concordam e confiam na política de imunização do Ministério da Saúde do país estão mais dispostos a vacinar seus filhos com as vacinas do calendário local^(19, 23, 38, 42, 43, 44, 48, 49, 57, 63, 70)^. A obrigatoriedade da vacinação infantil no país^(32, 41, 44, 46, 61)^ ou para o ingresso da criança na vida escolar auxilia na elevação das taxas de vacinação^(19, 20, 37, 81)^. Pais que apoiam governos^(49)^ e líderes que são a favor da vacinação infantil^(18)^ consideram a vacinação como importante para a saúde da criança. Pais que consideram o programa nacional de imunização local como completo^(71)^ não concordam em vacinar seus filhos com vacinas novas, enquanto que pais que percebem o calendário vacinal como rígido^(47, 61, 67)^ apresentam resistência em segui-lo. Pais que são contrários à lei da obrigatoriedade vacinal no país^(32, 44, 47, 68, 71)^ possuem descrença no governo, nas agências reguladoras^(32, 50)^ e no programa de imunização local^(^39,47,52,60,63,67,79,82,90^)^, não sendo adeptos à prática de vacinação infantil.

**Quadro 3:** Crenças dos pais sobre a vacinação infantil, 2024

**Crenças que inibem a vacinação infantil**	Vacinação como uma ação que causa dor e sofrimento^(53, 61, 74, 80)^, trauma^(38, 88)^, sobrecarga imunológica^(20, 26, 27, 32, 34, 40, 42, 44, 46, 49, 52, 67, 71, 75, 82, 84, 89, 90)^, riscos à saúde da criança^(19, 20, 21, 22, 23, 24, 25, 26, 27, 29, 31, 32, 33, 34, 37, 39, 40, 41, 42, 43, 45, 46, 47, 48, 49, 50, 51, 52, 53, 54, 56, 57, 59, 61, 62, 65, 66, 67, 68, 70, 71, 72, 73, 74, 75, 79, 82, 83, 84, 85, 86, 87, 88, 89, 80, 91, 92, 93)^ Descrença na efetividade da vacina^(23, 31, 35, 38, 40, 44, 46, 51, 59, 62, 65, 71, 75, 85, 88, 90, 91, 92)^ e na medicina ocidental^(23, 25, 38, 40, 51, 52, 60, 82, 85, 92)^. Insegurança, medo e incerteza em relação aos imunológicos (indústria farmacêutica, testes)^(18, 20, 23, 24, 31, 32, 33, 35, 38, 40, 41, 42, 43, 44, 51, 52, 56, 59, 61, 62, 67, 71, 75, 79, 83, 84)^ e ao processo de vacinação infantil^(37, 53, 79, 82, 85, 89)^. Crenças em falsas contraindicações para se vacinar^(18, 20, 21, 29, 33, 48, 49, 50, 53, 57, 61, 71, 75, 82, 84, 94)^. Crenças na imunidade adquirida naturalmente ser melhor do que a vacinação^(24, 39, 40, 42, 46, 50, 59, 62, 67, 71, 79, 84, 90)^. Experiências ruins de vacinação com filhos mais velhos^(20, 21, 29, 35, 37, 44, 46, 54, 55, 56, 61, 66, 68, 71, 83)^. Crenças de que seu filho é muito novo para ser vacinado, baseando-se em crenças culturais^(20, 32, 38, 39, 42, 47, 52, 59, 67, 71, 88)^ que a mãe foi imunizada na gestação^(88)^ e não necessita vacinar seu filho, que não está suscetível a contrair a doença imunoprevenível^(20, 32, 35, 62, 67, 71, 79,80,81,82, 83, 85, 86, 92)^, que as doenças imunopreveníveis não são comuns ou graves^(19, 23, 24, 33, 34, 43, 59, 61, 62, 65, 67, 68, 71, 79, 82, 83, 84, 85, 88, 90, 92)^, que podem controlar o curso delas confiando na qualidade do sistema de saúde local, e que crianças não imunizadas não representam perigo para a comunidade^(31)^. Sentimento de vergonha e crítica relacionado à pobreza pelas mães^(39, 49)^. Crenças em métodos alternativos para proteger as crianças contra as doenças imunopreveníveis^(79, 88)^, como amor^(67)^, amamentação^(20, 31, 38, 42, 47, 67, 71)^, alimentação^(25, 35, 67, 71, 82)^, homeopatia^(67)^, produtos fitoterápicos^(31, 35, 38, 50, 52)^ e práticas espirituais^(31)^. Crenças religiosas que afirmam que a vacinação causa malefícios para a saúde das crianças, considerando um pecado^(20, 21, 22, 25, 29, 35, 36, 39, 48, 50, 55, 61, 63, 64, 85)^
**Crenças que promovem a vacinação infantil**	Vacinar a criança é um dever^(42, 43, 67, 69, 74, 81)^ e um cuidado parental para poupar seus filhos da dor, do sofrimento e dos impactos de longo prazo na saúde em decorrência das doenças imunopreveníveis^(20, 21, 26, 37, 42, 53, 56, 69, 89)^. Vacinar protege tanto a criança do adoecimento grave e mortalidade^(18, 19, 24, 26, 28, 32, 34, 37, 42, 43, 45, 46, 48, 51, 53, 57, 58, 59, 65, 69, 70, 80, 81, 85, 86, 88, 90)^ quanto a comunidade^(19, 20, 46, 47, 52, 56, 79)^, e é uma decisão tomada sem estresse, onde os comportamentos da criança indicativos de sofrimento são encarados com naturalidade^(51, 69, 74)^ e as injeções múltiplas como necessárias e não prejudiciais^(27, 48, 51, 57, 83, 91)^. Crença religiosa de que a vacinação não é proibida pela religião^(48, 57)^ e é apoiada por líderes religiosos^(18)^ motiva os pais a vacinarem seus filhos. Experiências bem-sucedidas na vacinação de filhos mais velhos^(36, 46, 55, 70, 81, 85, 88)^ e a crença na vacinação como “norma cultural”^(42, 85)^ influenciam positivamente a vacinação dos filhos mais novos. Crença pessoal em considerar a vacinação como necessária por parte de pais que costumam viajar constantemente^(42)^.

As evidências sintetizadas no Quadro 2 permitem a identificação dos fatores mais citados pelos 77 estudos elegíveis como motivadores para a prática da vacinação infantil, sendo eles o conhecimento adequado sobre a vacinação infantil e a interação positiva com os profissionais de saúde. Esses determinantes possuem relação estreita entre si, já que as informações que os pais possuem sobre o imunizante e o processo de vacinação, a partir de atendimentos acolhedores, ricos em evidências científicas e abertos para esclarecimento de dúvidas com os profissionais de saúde, promovem a confiança familiar na imunização infantil que é traduzida em crenças promotoras que favorecem a atividade, conforme evidências do Quadro 3.

Alguns estudos não identificaram associação positiva entre o nível de conhecimento e a completude vacinal^(38, 47)^, ou seja, os pais alegaram não ter conhecimento sobre a temática apesar de apresentarem atitude favorável em relação à imunização infantil. Estudo caso-controle^(64)^ não encontrou diferenças significativas entre os pais dos dois grupos (vacinação completa e vacinação incompleta) em termos de conhecimento sobre vacinas, destacando que as variáveis mediadoras principais que aumentam a probabilidade de os pais completarem a vacinação de seus filhos são ausência de formação acadêmica, baixo nível de literacia comunicativa em saúde (capacidade de compreender o significado da informação médica, de modo a interagir com o ambiente médico), atitudes positivas elevadas em relação à vacinação, atitudes negativas fracas em relação à vacinação, atitudes negativas fracas em relação à vacinação obrigatória, considerando informações não oficiais de fontes que se opõem à vacinação como confiáveis. Outro artigo^(43)^ apresentou, como resultado da análise multivariada, que os determinantes da intenção dos pais tinham maior relação com a forma como os pais se sentiam sobre o seu conhecimento (ou seja, sentimento de estar suficientemente informado sobre a vacinação, conhecimento da importância de vacinar aos 2 meses) do que sobre o que eles objetivamente sabiam (conhecimento das doenças imunopreveníveis e das vacinas administradas aos 2, 4 e 6 meses de vida).

Entre os condicionantes que inibem a vacinação das crianças, nota-se prevalência dos fatores relacionados à interação limitada ou ineficiente com o profissional de saúde e as crenças, sejam elas religiosas, culturais, pessoais ou fundamentadas em experiências individuais que limitam a imunização da criança. Na medida em que os pais/responsáveis legais não são acolhidos pelos profissionais de saúde em seus questionamentos ou não recebem o suporte informacional necessário para a tomada de decisão a favor da vacinação, eles tendem a agir de acordo com suas crenças, especialmente centradas no risco do imunobiológico, e, com isso, podem adotar comportamentos que os afastam da prática da vacinação.

Os estudos evidenciam que, nessas situações, o comportamento de proteção parental está presente, mas praticado de forma equivocada: proteger *da* vacina e não *pela* vacina. Esse fenômeno perpassa, inclusive, pais que possuem altos níveis de escolaridade e conhecimento sobre a vacinação, porém com atitudes negativas em relação à imunização das crianças, demonstrando o poder que a crença exerce sobre o processo decisório da família^(47, 67)^.

## DISCUSSÃO

De acordo com os resultados presentes nesta revisão, a qualidade da interação entre pais ou responsáveis legais e profissionais de saúde é o principal fator mediador no processo de tomada de decisão em relação à vacinação infantil, segundo a percepção dos participantes.

Esse achado encontra-se em conformidade com outro estudo, no qual constatou-se que muitos pais atribuem a não vacinação de suas crianças (recusa ou atraso) à forma como são tratados pelos profissionais que atuam nos consultórios e nos serviços de vacinação^(95)^. Conforme revisão de literatura (*overview*), a interação entre pacientes e prestadores de serviços é a pedra angular para manter a confiança na vacinação. O conhecimento e as atitudes desses profissionais sobre as vacinas já demonstraram ser cruciais na sua própria adesão à vacina, na sua intenção de recomendá-la e na adesão à vacina dos seus pacientes ^(96)^.

Por esse motivo, encontrar um profissional cético pode mudar fortemente a opinião das pessoas ou reforçar a ideia de que a vacinação não é segura, especialmente entre aqueles que já a recusam. A necessidade de fortalecer a confiança nas vacinas acompanha a necessidade de melhorar as habilidades de comunicação com os demandantes de cuidado (crianças, pais, famílias e comunidades)^(95)^.

Como os profissionais de saúde desempenham um papel fundamental na proteção e promoção da saúde da criança e têm o dever de informar as pessoas sobre as vacinas e os riscos decorrentes de uma cobertura vacinal que não atinge as metas preconizadas, é essencial o investimento em educação permanente, comunicação e literacia em saúde para esses profissionais^(95)^.

O fortalecimento da Atenção Primária está, entre outros fatores, no reconhecimento de que a produção de saúde se faz entre pessoas e de que é preciso aprimorar o modo de relação estabelecido entre os serviços de Atenção Primária à Saúde (APS), como a vacinação, com os seus usuários. É essencial que os serviços sejam facilmente acessíveis à população, que o usuário esteja no centro da atenção e que a orientação às necessidades em saúde das comunidades seja a base da organização dos serviços^(97)^. Dessa forma, o enfermeiro, ao identificar os diversos nós críticos que envolvem o processo e ao planejar ações efetivas e direcionadas, para a superação do problema das baixas coberturas vacinais, destaca a importância da imunização como medida básica para o controle de doenças imunopreveníveis, promovendo o sucesso dessa estratégia na saúde da comunidade como ator principal no território.

A confiança nas vacinas e a percepção do risco da vacina estão diretamente relacionadas à hesitação vacinal. Estudo com o objetivo de avaliar a hesitação vacinal materna e seus fatores associados identificou que variáveis como renda familiar elevada, bom relacionamento com os profissionais de saúde, disposição para aguardar a vacinação e campanhas foram associadas a uma maior confiança e menor percepção do risco das vacinas. Por outro lado, o atraso deliberado ou a decisão de não vacinar a criança e a experiência anterior com reações adversas à vacina foram associadas a menor confiança e maior percepção do risco das vacinas. Este estudo também destaca que os prestadores de cuidados de saúde, especialmente os enfermeiros, desempenham um papel relevante para abordar a hesitação vacinal, orientando a vacinação através de um relacionamento confiável^(98)^.

Apesar da notória presença da enfermagem atuando na coordenação e implementação do serviço de vacinação na APS, nesta revisão, observou-se o limitado reconhecimento desse profissional como fonte de informação em saúde, havendo o direcionamento à categoria médica (pediatras) quando menciona-se a classe de profissionais de saúde.

O sistema de crenças (culturais, religiosas, pessoais), assim como as experiências individuais dos pais ou responsáveis legais, destacam-se nesta revisão como um significativo determinante na atitude de vacinar, adiar ou recusar a vacinação.

O Modelo de Crenças em Saúde (HBM, sigla em inglês) pode auxiliar na compreensão dos motivos que levam os pais e/ou responsáveis pelas crianças a aceitarem ou não o imunobiológico à medida que a ação relacionada à saúde depende da ocorrência simultânea de três fatores: motivação suficiente para tornar as questões de saúde salientes ou relevantes; crença na susceptibilidade a um problema de saúde sério ou suas sequelas, conhecida como ameaça percebida; e a crença de que seguir uma recomendação de saúde específica seria benéfico na redução da ameaça percebida a um custo subjetivamente aceitável. O custo refere-se às barreiras percebidas que devem ser superadas para seguir a recomendação de saúde^(7)^.

A interpretação popular do risco nem sempre se fundamenta em uma abordagem racional, mas sim em uma abordagem de “incertezas e ambiguidades”, em que as incertezas permanecem mesmo diante da evidência científica^(99)^.

Assim, a adesão à vacinação está sujeita ao imaginário social, que influencia sobremaneira a propensão de um determinado grupo de se vacinar ou não. Há vários fatores que afetam tal decisão, como a confiança na importância, segurança e eficácia das vacinas, bem como a compatibilidade com os valores religiosos do indivíduo. Tais dados foram constatados nos países com maiores percentuais de concordância nos quesitos que as vacinas são seguras, importantes e eficazes, uma vez que apresentaram maior percentual de relatos de terem vacinado seus filhos^(100)^. Somam-se a isso a confiança da população nas vacinas e a sua adesão, fruto da convivência recente com doenças imunopreveníveis como ocorrido na Africa, América Latina e Índia^(101)^.

Outro aspecto que tem íntima relação com as crenças é a hesitação vacinal, definida como o atraso na aceitação ou recusa das vacinas, apesar da disponibilidade de serviços de vacinação^(5)^. Uma atitude hesitante em relação à vacinação infantil significa que alguns pais têm dúvidas sobre os benefícios das vacinas, preocupando-se com sua segurança e questionando a necessidade delas^(102)^. Por conseguinte, os elevados índices de hesitação vacinal acarretam a baixa demanda de vacinas e a cobertura vacinal^(5)^.

Em consonância com os achados desta revisão, estudo que analisou, nas produções científicas, os motivos que levam pais e familiares de crianças à hesitação vacinal no contexto do controle das doenças imunopreveníveis, encontrou que esses estavam relacionados ao des(conhecimento) sobre as vacinas (*fake news,* medo de eventos adversos, subestimação da letalidade das doenças imunopreveníveis) e à in(decisão) e ao estilo de vida (hábitos saudáveis, medicina alternativa e religião^(103)^.

Esta revisão mapeou como crenças inibidoras, entre outras, a sobrecarga imunológica, os riscos à saúde da criança, a descrença na efetividade da vacina, a insegurança, o medo e a incerteza em relação aos imunológicos (indústria farmacêutica e testes). Essas evidências são corroboradas por um inquérito vacinal realizado em 28 países da Europa em 2019, onde se evidenciou que aproximadamente um décimo da população europeia considerava que as vacinas não são rigorosamente testadas antes da autorização. Um terço acreditava que as vacinas podem sobrecarregar ou enfraquecer o sistema imunológico e que podem causar a doença contra os quais protegem, e quase metade acreditava que as vacinas podem causar efeitos secundários graves. Além disso, identificaram-se três configurações de crenças em relação à eficácia, segurança e utilidade da vacina: o tipo hesitante (11%) é definido pela percepção de que as vacinas são ineficazes; o tipo confiante (59%) é definido por crenças de que as vacinas são eficazes, seguras, bem testadas e úteis; e o tipo *tradeoff* (conflito de escolha) (29%) combina crenças de que as vacinas são eficazes, bem testadas e úteis, com percepções de provável dano ou risco. Os tipos que confiam na vacina e os do tipo *tradeoff* têm históricos de vacinação semelhantes, indicando o papel significativo de outros fatores além das crenças na indução do comportamento^(104)^.

Estudo que teve como objetivo analisar as representações sociais de brasileiros hesitantes sobre vacinação contra COVID-19 concluiu que as representações apreendidas demonstram a dificuldade deles em discernir sobre a confiabilidade das informações e um imaginário social de dúvidas e incertezas. Assim, as representações sociais sobre um determinado objeto ou fenômeno são visões de mundo impregnadas de valores socioculturais e crenças que se constroem ao longo de uma trajetória de vida e são capazes de impactar as práticas sociais de saúde^(99)^.

Na vacinação infantil, as representações são também determinantes, devido à diversidade e multiplicidade de doses dos imunobiológicos recomendados no primeiro ano de vida e à representação dessa prática para os pais como de sobrecarga e de maiores riscos a efeitos adversos. Nesse cenário, o viés de omissão dos pais, definido como ser mais avesso aos riscos associados a uma ação — tomar uma vacina “insegura” — do que aos riscos associados à inação — correr o risco de contrair uma doença imunoprevenível, tem papel relevante nas atitudes de rejeição ou adiamento da vacinação dos indivíduos^(96)^.

Os achados da revisão se relacionam com o modelo dos 3Cs^(105)^ proposto pela OMS, especialmente no que se refere ao componente das crenças parentais (Quadro 3), pois a complacência (não perceber as doenças como de alto risco e a vacinação como necessária) e a confiança (falta de confiança na segurança e eficácia das vacinas) são os domínios mais influenciados por elas, resultando em comportamentos hesitantes por parte dos pais. Nesse âmbito, portanto, a educação em saúde pode atuar de forma eficaz visando desmitificá-las, promovendo conhecimentos claros e baseados em evidências científicas^(95)^.

### Limitações do estudo

A presente revisão de escopo apresenta como limitações a não inclusão da literatura cinzenta, a restrição de idiomas (inglês e português) na busca das fontes de evidências e a não inclusão de estudos sobre vacinação de campanha. Destaca-se a importância de estudos com populações rurais e com crianças portadoras de comorbidades, considerando que não foram as populações consideradas como foco desta revisão.

### Contribuições para a área da enfermagem, saúde ou política pública

Esta revisão aponta como recomendações práticas a capacitação dos profissionais de saúde quanto ao conhecimento científico sobre imunização e as competências comunicacionais com a família no contexto da vacinação infantil, com o objetivo de diminuir receios da população e aumentar a confiança, ao exercerem a educação em saúde baseada em evidências. O aconselhamento oportuno dos pais, a ênfase na vacinação na idade recomendada e a investigação do estado de vacinação podem ser realizados pelos prestadores de cuidados de saúde em qualquer ambiente localizado em um estabelecimento de saúde, e a sensibilização dos pais sobre o assunto deve ser feita, idealmente, no início da gestação. Quanto aos calendários de vacinação alternativos, os prestadores de cuidados de saúde precisam ser cautelosos e discutir os riscos associados ao atraso da vacinação ao negociar com os pais sobre quando e como as vacinas infantis podem ser completadas.

Destaca-se a importância da inserção e do aprofundamento da temática da imunização no sistema educativo, vislumbrando um potencial impacto na saúde individual e comunitária, posicionando os indivíduos para estarem mais bem preparados para gerir a informação sobre a sua própria saúde ao longo da vida.

Os achados sugerem recomendações relacionadas a novas pesquisas envolvendo a figura do(a) enfermeiro(a) no processo de vacinação infantil, sobre mídia e campanhas educacionais personalizadas e bem elaboradas, bem como trabalhos científicos em áreas remotas e de difícil acesso. Recomenda-se também o desenvolvimento de estudos com vistas ao mapeamento das percepções e crenças dos pais em relação à vacinação infantil na estratégia de campanha, para que se possa estabelecer uma comparação entre as duas formas de proteção da saúde coletiva quanto aos seus determinantes no processo decisório dos responsáveis e significados da prática para essa população.

Devido à vasta gama de determinantes individuais e sociais que impulsionam ou dificultam a aceitação das vacinas no público pediátrico, recomenda-se a realização de pesquisas locais para a identificação dos principais fatores que afetam o êxito do programa de imunização em articulação com as características e particularidades regionais, já que a revisão tem o objetivo de sintetizar essas evidências de forma global.

## CONCLUSÕES

Esta revisão de escopo mapeou um conjunto de evidências sobre as percepções, crenças, conhecimentos e atitudes dos pais ou responsáveis legais sobre a vacinação infantil de rotina, bem como seus fatores relacionados. Na percepção dos pais, os principais determinantes para a tomada de decisão em relação à vacinação da criança são: a interação com os profissionais de saúde; as crenças (culturais, religiosas, pessoais) e experiências individuais; os conhecimentos sobre vacinação infantil; o acesso aos serviços de saúde de forma universal e gratuita; a dinâmica e padrões de organização familiar; a estrutura familiar; as interações sociais; e as políticas públicas sociais e de saúde que envolvem os programas de vacinação. Esses determinantes influenciam o significado e o comportamento parental.

As evidências representativas da percepção dos pais permitiram a identificação das crenças tanto inibidoras, geradoras de sofrimento e medo, quanto promotoras, pautadas no dever e cuidado parental, determinantes para a adesão à vacinação infantil. Esse conhecimento é suporte para a prática da enfermagem centrada nas crenças e necessidades subjetivas e singulares dos pais ou responsáveis legais de crianças menores de 5 anos na abordagem da hesitação vacinal.

## Data Availability

https://doi.org/10.48331/scielodata.VL93JN
